# Pinecone-Inspired Water-Responsive Curling Adhesive Conduit for Peripheral Nerve Repair

**DOI:** 10.34133/cbsystems.0556

**Published:** 2026-03-27

**Authors:** Xiaolei Guo, Jinwei Li, Hongyu Xu, Shengrong Long, Junhong Li, Ao Wang, Wenkai Liu, Fan Zhang, Zhen Li, Feng Luo, Jiehua Li, Yanchao Wang, Hong Tan, Ting Lan

**Affiliations:** ^1^ College of Polymer Science and Engineering, National Key Laboratory of Advanced Polymer Materials, Med-X Center for Materials, Sichuan University, Chengdu 610065, China.; ^2^ Department of Neurosurgery, West China Hospital, Sichuan University, Chengdu, Sichuan 610000, China.; ^3^ Brain Research Center, Zhongnan Hospital of Wuhan University, Wuhan University, Wuhan 430071, China.; ^4^ Department of Thoracic Surgery, Tongji Hospital, Tongji Medical College, Huazhong University of Science and Technology, Wuhan 430030, China.; ^5^ Department of Pathology, Sichuan Clinical Research Center for Cancer, Sichuan Cancer Hospital & Institute, Sichuan Cancer Center, University of Electronic Science and Technology of China, Chengdu 610000, China.

## Abstract

Neural guidance conduits represent a promising approach for treating peripheral nerve injuries. However, challenges remain, including the complexity of microsuturing, potential iatrogenic damage, and variations in nerve dimensions. Inspired by the deformation of pinecone scales upon water absorption, this study developed a water-responsive self-curling adhesive conduit to achieve adaptive wrapping and suture-free repair for peripheral nerve injury. The self-curling film (PU/PGA_*X*_) was composed of hydrophilic γ-polyglutamic acid (PGA) and hydrophobic polyurethane (PU). Differential swelling of these components in water drives the autonomous curling of the membrane into a tubular structure. Coating the self-curling film with a PU adhesive emulsion endows the material with adhesion functionality. The resulting self-curling adhesive conduit can adaptively wrap around nerve tissue and achieve adhesion fixation, providing a biomimetic scaffold for nerve regeneration. Furthermore, the PGA-loaded conduit exhibits excellent biocompatibility and effectively promotes peripheral nerve regeneration. This self-curling adhesive conduit offers straightforward operability and demonstrates marked repair efficacy, indicating substantial potential for applications in nerve repair.

## Introduction

Peripheral nerve injury (PNI) is a common form of neurological injury that can occur due to various causes [1,2]. This type of injury can lead to sensory and motor dysfunction, severely impairing bodily function and activities of daily living [3]. The inherently complex structure and function of human nerve tissue make autologous repair difficult. For long-distance peripheral nerve injuries, autologous nerve grafts or allogeneic nerve grafts are typically employed [4,5]. However, the utility of autologous nerve grafting is limited by factors including limited tissue availability, donor site morbidity, functional loss, and potential mismatches in tissue size and structure [6,7]. Thus, novel strategies are required to bridge the injured nerve and facilitate nerve regeneration and functional recovery [8].

Recent advances in tissue engineering have opened new avenues for peripheral nerve repair, with nerve guidance conduits (NGCs) emerging as a highly promising alternative strategy [9,10]. These tubular scaffolds provide directional guidance for axonal regeneration by mimicking the physical instructive role of the nerve extracellular matrix (ECM) [11–13]. Current manufacturing techniques for artificial NGC, including solution casting/dipping, injection molding, melt extrusion, and 3-dimensional (3D) printing, primarily aim to replicate the basic tubular structure of nerves [14–16]. However, conduits fabricated using these methods typically exhibit homogeneous structural features, in stark contrast to the inherently variable diameters and irregular geometries of natural nerve tissue. Consequently, these conduits often fail to achieve adaptive wrapping contact with the epineurium. This structural mismatch may severely compromise nerve repair outcomes. Furthermore, the fixation of these artificial conduit scaffolds to nerve stumps typically relies on conventional microsuturing [17,18]. This approach not only demands advanced microsurgical skills but also carries risks of complications such as inflammation, fibrosis, scar tissue formation, and axonal misdirection. Moreover, in nerve anastomosis and repair operations where the operating space is extremely limited, such as in the brain, microsuture becomes even more challenging. Tissue adhesives present a potential alternative to traditional suturing [19]. Therefore, developing nerve conduits endowed with both adhesive fixation capability and self-adaptive wrapping functionality represents an ideal material solution for repairing peripheral nerve injuries.

The pinecone is a type of plant fruit, and its scales change from being open to closed as the humidity increases [20,21]. This is mainly due to the difference in water absorption and expansion between the inner and outer layers of the pinecone scales [22]. The outer layer is more prone to absorbing water and expanding, while the inner layer does not change easily [23]. This difference alters the curvature of the pinecone and causes it to close in a humid environment. Inspired by the deformation of the pinecone scales, this study prepared a water-responsive self-curling adhesive conduit by regulating the distribution difference of hydrophilic and hydrophobic components at the top and bottom of the film for repairing PNI. To improve the water responsiveness of the material, the hydrophilic component γ-polyglutamic acid (PGA) was added to the polyurethane (PU) emulsion containing hydrophobic polycaprolactone (PCL) and dried to form a film. The hydrophilic PGA rapidly absorbs water and expands, while the hydrophobic PU forms a structural support layer. The swelling difference produced after water absorption can drive the material to transform from a flat state to a tubular structure. The PU emulsion adhesive coating was applied to the self-curling film for functional integration. This adhesive layer can achieve fixed adhesion between the conduit and the nerve tissue, avoiding microsuture. The PU/PGA_10_ patch containing 10% PGA exhibits rapid curling dynamic characteristics and can quickly curl into a tubular structure in physiological saline and achieve adhesion and fixation of the severed nerve. The PU/PGA_10_ adhesive conduit in the rat sciatic nerve injury model can promote macrophage polarization toward the M2 pro-healing type and promote the regeneration and repair of the injured nerve in rats 10 weeks after surgery. This water-responsive self-curling adhesive conduit has great potential for clinical nerve repair applications.

## Materials and Methods

### Preparation of PU emulsion

Polyethylene glycol 1450 (2.46 g) and 16.6 g of poly(caprolactone) diol 2000 were added to a 250-ml 3-necked flask. The mixture was heated to 95 °C and dehydrated under vacuum for 2 h. Subsequently, 4.89 g of isophorone diisocyanate and 0.1% bismuth-based catalyst were added, and the reaction proceeded at 80 °C for 2 h. The temperature was then lowered, acetone was added, and 2.76 g of quaternary ammonium salt diol chain extender (Q12) was introduced to dissolve. Chain extension was carried out at 55 °C for 3 h. After cooling the reaction mixture to room temperature, 0.37 g of L-lysine (Lys) dissolved in water was added and stirred for 5 min. The product was then poured into water and stirred for emulsification for 1 h to obtain the PU emulsion. Acetone was removed from the resulting emulsion by rotary evaporation to yield the pure PU emulsion. The detailed synthetic route is shown in Fig. S3.

### Preparation and characterization of self-curling films and patches

An aqueous PGA solution (10 wt%) was mixed with a PU emulsion (10 wt% solid content) at specific mass ratios. The mixture was poured into glass Petri dishes and rapidly dried in a forced-air oven at 50 °C for 48 h to obtain PU/PGA_*X*_ films, where *X* denotes the mass percentage of PGA in the composite film. PU adhesive emulsions (QLPU_75_) were synthesized following the method previously reported by our group [24]. This adhesive emulsion was uniformly coated onto the surface of the PU/PGA_*X*_ films. The coated films were then dried in a forced-air oven at 50 °C for 24 h, yielding self-curling adhesive patches, designated as PU/PGA_*X*_ patches. The curling rate and angle of the PU/PGA_*X*_ patch can be adjusted by controlling the PGA content and the film thickness. Infrared spectra of both sides of the self-curling film were obtained within the range of 400 to 4,000 cm^−1^ using a Nicolet iS 50 spectrometer. The elemental distribution on both sides of the self-curling film was analyzed by x-ray photoelectron spectroscopy (XPS). The PU/PGA_10_ and PU films were immersed in water for 5 d, taken out and placed in a −20 °C freezer. After freezing, they were lyophilized using a freeze dryer. The micromorphology of the top and bottom surfaces of the films was then examined by scanning electron microscopy (SEM). The patches were cut into dumbbell-shaped specimens, and tensile tests were performed using a universal testing machine. The adhesive strength of PU/PGA_10_ patches was tested through a shear lap model.

The PU/PGA_*X*_ patch was immersed in water, and the curling angle at different times was recorded using a camera to calculate the curling curvature [25,26]. In Fig. S4, *L* represents the patch length, and *θ* represents the bending angle, curvature: *k* = (*Π* × *θ*)/(180° × *L*). PU/PGA_10_ self-curling patches with different thicknesses were prepared by controlling the mass of the mixed solution, and their curling curvatures were calculated.

### In vitro cell experiments

Rat Schwann cells (RSC96 cells) were used to study the cytocompatibility of PU/PGA_10_ patches. The Cell Counting Kit-8 (CCK-8) assay was employed to detect cell viability after coculture with extracts from PU patches and PU/PGA_10_ patches. RSC96 cells were seeded in 96-well plates, treated with patch extracts for 2 d, stained using the Calcein-AM/PI reagent kit, and photographed under a fluorescence microscope to evaluate cell growth. After coculturing RSC96 cells with PU/PGA_10_ patches and PU patches for 1 d, cytoskeleton staining was performed using rhodamine-labeled phalloidin. Cells were observed and images were acquired under a confocal laser scanning microscope for analysis. The cell scratch assay was used to detect the effect of extracts from PU/PGA_10_ patches and PU patches on the migration ability of RSC96 cells. Microscope images at 0 and 48 h were used to monitor cell migration. The Transwell migration assay was employed to detect the effect of extracts from PU/PGA_10_ patches and PU patches on the migration ability of RSC96 cells. Migrated cells were fixed with paraformaldehyde, stained with crystal violet, and photographed under a microscope. Images were processed and counted using ImageJ software to calculate the number of migrated cells.

### Self-curling patch for repair of rat sciatic nerve injury

The animal experiments were conducted according to the official guideline of the Institutional Animal Care and Use Committee of China, and protocols were approved by the Laboratory Animal Welfare and Ethics Committee of West China Hospital Sichuan University (Permit Number: 20240221049). After anesthetizing Sprague-Dawley (SD) rats (female, body weight 180 to 220 g) and placing them in lateral recumbency, the skin and muscles were incised using surgical tools following iodine disinfection to expose the right sciatic nerve. An 8-mm segment of the right sciatic nerve was excised to create a defect. The rats were randomly divided into 4 groups: the PU/PGA_10_ patch group, the PU patch group, the autograft group, and the control group. In the PU/PGA_10_ patch group, physiological saline was used to induce self-curling adhesion for bridging the nerve ends. In the PU patch group, the patch was manually wrapped around the nerve ends. In the autograft group, the excised sciatic nerve segment was reversed and sutured back into the defect site. In the control group, no further treatment was performed after nerve resection. Meloxicam was injected every 24 h for 3 d after surgery to alleviate pain. At 2 and 10 weeks postsurgery, motor function was evaluated using the sciatic function index (SFI). Rats were euthanized at 2 and 10 weeks postoperation to harvest the right sciatic nerve and bilateral gastrocnemius muscles for repair effect evaluation.

### Analysis of rat walking trajectory

At 2 and 10 weeks postoperation, motor function was assessed using the SFI to evaluate repair outcomes. Rats were allowed to walk freely in a transparent glass channel, and a camera placed beneath the glass was used to capture images of their walking tracks. SFI was calculated using Eq. (1):

$$\begin{array}{c} \mathrm{SFI}=109.5\times \left(\mathrm{ETS}-\mathrm{NTS}\right)/\mathrm{NTS}-38.3\\ \;\times \left(\mathrm{EPL}-\mathrm{NPL}\right)/\mathrm{NPL}+13.3\times \left(\mathrm{EIT}-\mathrm{NIT}\right)/\mathrm{NIT}-8.8\;\; \end{array}$$ (1)

In the formula, E and N represent the experimental (surgical) side and the normal side, respectively; PL is the print length, TS is the toe spread, and IT is the intermediary toe spread. The SFI value ranges from 0 (normal function) to 100 (complete impairment).

### Gastrocnemius muscle analysis

At 2 and 10 weeks postoperation, rats were euthanized, and the gastrocnemius muscles were harvested, photographed, and weighed. The muscles were then fixed in 4% paraformaldehyde solution for Masson staining. The proportion of muscle fibers and the diameter of muscle fibers on the surgical side were calculated and measured using ImageJ software.

### Histological analysis of regenerated nerves in rats

At 10 weeks postoperation, rats were euthanized, and the skin and muscles on the surgical side were incised to expose the regenerated nerves. The middle segment of the regenerated nerve was collected and fixed in 4% paraformaldehyde solution. Subsequently, immunofluorescence double staining for S100 and NF200 was performed to label regenerated axons and Schwann cells. Vascularization was marked by CD31 and α-SMA immunofluorescence staining. The percentage of positively stained areas was calculated using ImageJ software.

### TEM analysis of regenerated nerves in rats

At 10 weeks postoperation, the regenerated nerves from the surgical side were collected and fixed in electron microscopy fixative. After dehydration, embedding, and sectioning, the sections were double-stained with uranyl acetate and lead citrate. The ultrastructure of the regenerated nerves was observed using transmission electron microscopy (TEM).

### Analysis of inflammation at 2 weeks post-nerve injury repair

At 2 weeks postoperation, rats were euthanized, and the injured nerves from the surgical side were collected and fixed in 4% paraformaldehyde solution. Subsequently, immunofluorescence double staining for CD68/CD86 and CD68/CD206 was performed to evaluate the immune microenvironment at the injury site during the early stage of repair. The percentage of positively stained areas was calculated using ImageJ software.

### Statistical analysis

All data were calculated as mean ± standard deviation and analyzed using statistical software (IBM SPSS). A one-way analysis of variance was employed for analysis. *P* value less than 0.05 was considered significant, *P < 0.05, **P < 0.01, ***P < 0.001.

## Results and Discussion

### Preparation of PU/PGA_*X*_ films and evaluation of self-curling behavior

**Fig. 1.**
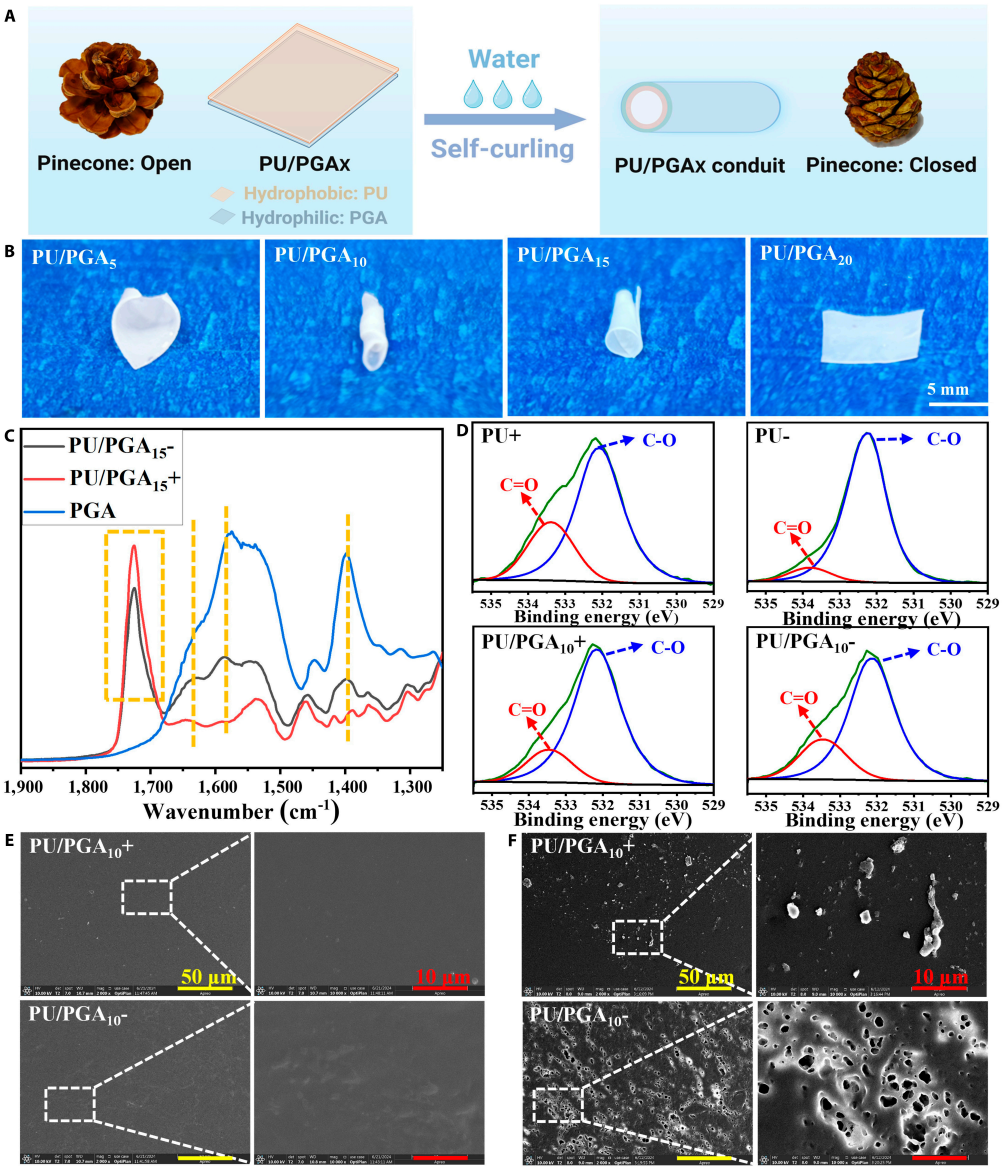
(A) Shape changes and schematic diagrams of pinecones and polyurethane (PU)/γ-polyglutamic acid (PGA_*X*_) before and after soaking in water. (B) Shape of PU/PGA_*X*_ film after soaking in water. (C) ATR-FTIR spectra of top (+) and bottom (−) of the PU/PGA_15_ film. (D) O1s x-ray photoelectron spectroscopy (XPS) spectra of PU and PU/PGA_10_ films. Scanning electron microscopy (SEM) images of the top (+) and bottom (−) of the PU/PGA_10_ film before (E) and after (F) water immersion.

The waterborne PU containing PCL soft segments and quaternary ammonium diol chain extender (Q12) was synthesized as a hydrophobic component following the route outlined in Fig. S3. Quaternary ammonium salts have an amphiphilic structure, consisting of a hydrophilic quaternary ammonium head group and a hydrophobic long alkyl chain [27]. This unique structure enables them to function as surfactants, facilitating migration and enrichment at the air–liquid interface [24,28]. The PU emulsion was mixed with varying ratios of PGA and subsequently dried rapidly in a blowing oven set at 50 °C. The quaternary ammonium salt within the PU functioned as a surfactant, causing the PU to migrate to the surface. This process led to the formation of an asymmetric film, featuring a more hydrophobic PU layer on top and a more hydrophilic PGA layer at the bottom (Fig. 1A). The asymmetric distribution of hydrophilic and hydrophobic components causes it to deform like a pinecone when exposed to water. The PU/PGA_*X*_ films (where *X* denotes the mass ratio of PGA) were immersed in water to observe their behavioral changes. As shown in Fig. S5 and Fig. 1B, PU/PGA_5_, PU/PGA_10_, and PU/PGA_15_ all demonstrated upward self-coiling behavior (Movie S1). This phenomenon can be attributed to the hydrophilic nature of PGA and the hydrophobic property of PU within the PU/PGA_*X*_ composite structure. Upon soaking in water, the hydrophilic PGA region at the bottom of the film rapidly expanded, while the hydrophobic PU region at the top absorbed minimal water and exhibited negligible volume change. This disparity in expansion between the 2 sides of the film led to uneven stress distribution, causing the film to curl toward the side with less swelling.

### Characterization of PU/PGA_*X*_ films on both sides

The 2-sided properties of PU/PGA_*X*_ self-coiled films were characterized using attenuated total reflectance (ATR) surface infrared spectroscopy, XPS, and SEM. As shown in Fig. 1C, the PU/PGA_15_ film exhibits a C=O peak near 1,725 cm^−1^, which is derived from PCL and carbamate in PU [29]. Notably, the intensity of the C=O peak at the top of the PU/PGA_15_ film is higher than that at the bottom, indicating that PU is more present at the top. Conversely, the characteristic peaks of PGA at the bottom of the PU/PGA_15_ film, around 1,581 cm^−1^ (N-H bending vibration in amide bond) and around 1,400 and 1,630 cm^−1^ (carboxyl group symmetric and asymmetric stretching vibration) [30–32], are more pronounced than those at the top, suggesting more distribution of PGA at the bottom. Table S3 and Fig. 1D present the fitting results for the percentage of each element and the split peak analysis of the O1s spectrum in the top and bottom XPS spectra of PU film and PU/PGA_10_ film, respectively. The O1s spectrum was divided into 2 distinct peaks, corresponding to C=O and C–O [33,34]. Specifically, the C=O oxygen content at the top and bottom of the PU film constitutes 6.14% and 2.21% of the total elements, respectively. After the addition of PGA, the O=C oxygen content in the PU/PGA_10_ film decreased to 4.46% at the top and increased to 5.46% at the bottom. This phenomenon can be attributed to the fact that PGA is predominantly distributed at the bottom of the PU/PGA_10_ film, where the O=C oxygen primarily originates from the carboxyl group within PGA. In addition, Table S1 shows that N^+^ content is 1.07% at the top of the PU/PGA_10_ film, which is higher than 0.85% at the bottom. N^+^ is derived from the quaternary ammonium salt in PU [35]; this finding suggests that PU is more concentrated at the top of the PU/PGA_10_ film compared to the bottom.

The surface morphology of the top and bottom of the PU/PGA_10_ film was observed by SEM. As shown in Fig. 1E, no obvious differences were observed between the top and bottom surfaces of the PU/PGA_10_ film prior to water soaking. After soaking in water for 5 d, Fig. 1F reveals the formation of noticeable holes at the bottom of the PU/PGA_10_ film. This phenomenon is attributed to the hydrophilic PGA gradually dissolving out of the film, whereas the PU structure remains stable and insoluble in water. In contrast, no obvious pore formation was detected on the top surface of the PU/PGA_10_ film. The SEM results clearly demonstrated that the hydrophilic PGA was predominantly located at the bottom of the film. The 2-sided characterization further revealed an asymmetric structure in the PU/PGA_10_ films, with hydrophilic PGA concentrated on the bottom surface and PU dominating the upper surface.

### Analysis of the film formation process

**Fig. 2.**
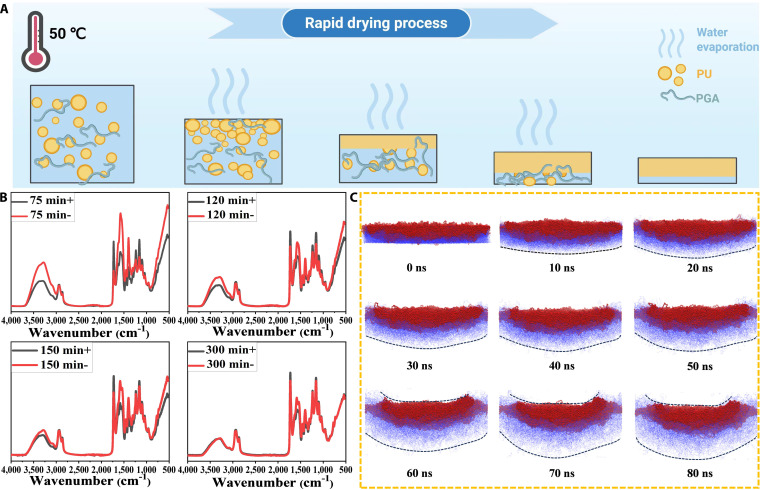
(A) Schematic diagram of rapid drying and film formation of PU/PGA_*X*_ solution. (B) Top (+) and bottom (−) ATR-FTIR spectra of PU/PGA_10_ mixed solution dried for different times. (C) Coarse-grained molecular dynamics simulation of swelling and state changes of different components of film after water bubble (red: hydrophobic, blue: hydrophilic).

The hydrophilic and hydrophobic components of the PU/PGA_10_ mixed solution formed an asymmetric distribution after rapid drying. As shown in Fig. 2A, upon exposure to high-temperature air, the water on the surface of the solution evaporates quickly, enriching the solute on the surface and forming a layer of shell cortex. The presence of a quaternary ammonium surfactant ensures that the top shell is predominantly composed of PU. This shell delays the evaporation of water from the underlying layers, causing the hydrophilic PGA to accumulate in the residual water at the bottom. As the water slowly diffuses and evaporates from the bottom, more PGA remains concentrated there, resulting in an asymmetric film with higher concentrations of PGA at the bottom and PU at the top. The mixed solution of PU/PGA_10_ was poured into the Petri dish and placed in the air oven at 50 °C. The process of drying the solution and forming the film was monitored by ATR surface infrared. At 75 min, a continuous solid “dry skin” layer formed on the surface of the solution. Beneath this layer, water that had not fully evaporated remained as a liquid paste. This indicated that by this time, a continuous solid film layer had formed on the top of the sample, while the bottom still retained its liquid paste consistency. As shown in Fig. 2B, the infrared spectrum at the bottom exhibited prominent stretching vibration peaks of water molecular between 3,200 and 3,600 cm^−1^ at 75 min [36]. In contrast, the corresponding characteristic peaks at the top were nearly absent, aligning with the experimental observations. With the extension of drying time, the intensity of the characteristic peak of water molecules in the bottom spectrum gradually decreased, indicating that the bottom water gradually completed the diffusion and evaporation (Fig. 2B). However, the PGA characteristic peak intensity at the bottom of PU/PGA_10_ around 1,581, 1,400, and 1,630 cm^−1^ remained higher than that at the top until a dry PU/PGA_10_ film was formed at 300 min. During this dynamic process, the hydrophilic PGA components tended to accumulate more in the bottom layer containing residual water, thereby forming an asymmetric distribution as the water continued to volatilize.

### Study on the mechanism of self-curling

The behavior of self-coiled films in an aqueous environment was simulated by coarse-grained molecular dynamics. As shown in Fig. 2C, the film remains flat at the initial stage, and the hydrophilic component at the bottom (blue) begins to absorb water and diffuse swelling over time, while the swelling restricted structure of PU at the top (red) remains relatively stable. The difference in swelling between the top and bottom of the film starts to drive the material to automatically curl up on the upward surface at 60 ns, and this result is consistent with the self-coiling behavior observed experimentally. Although molecular dynamics simulations are difficult to match the actual situation of the patches in terms of time and length scales, they can assist in verifying the movement and behavioral changes of hydrophilic and hydrophobic components when exposed to water. When hydrophilic and hydrophobic components form an asymmetric distribution at the top and bottom of the membrane, the bottom hydrophilic group swells rapidly after the film soaks in water, while the top PU remains rigid and low swelling, and the swelling difference on both sides of the membrane drives the material to automatically curl to the low swelling side.

### Preparation and characterization of PU/PGA_*X*_ self-curling adhesive conduits

**Fig. 3.**
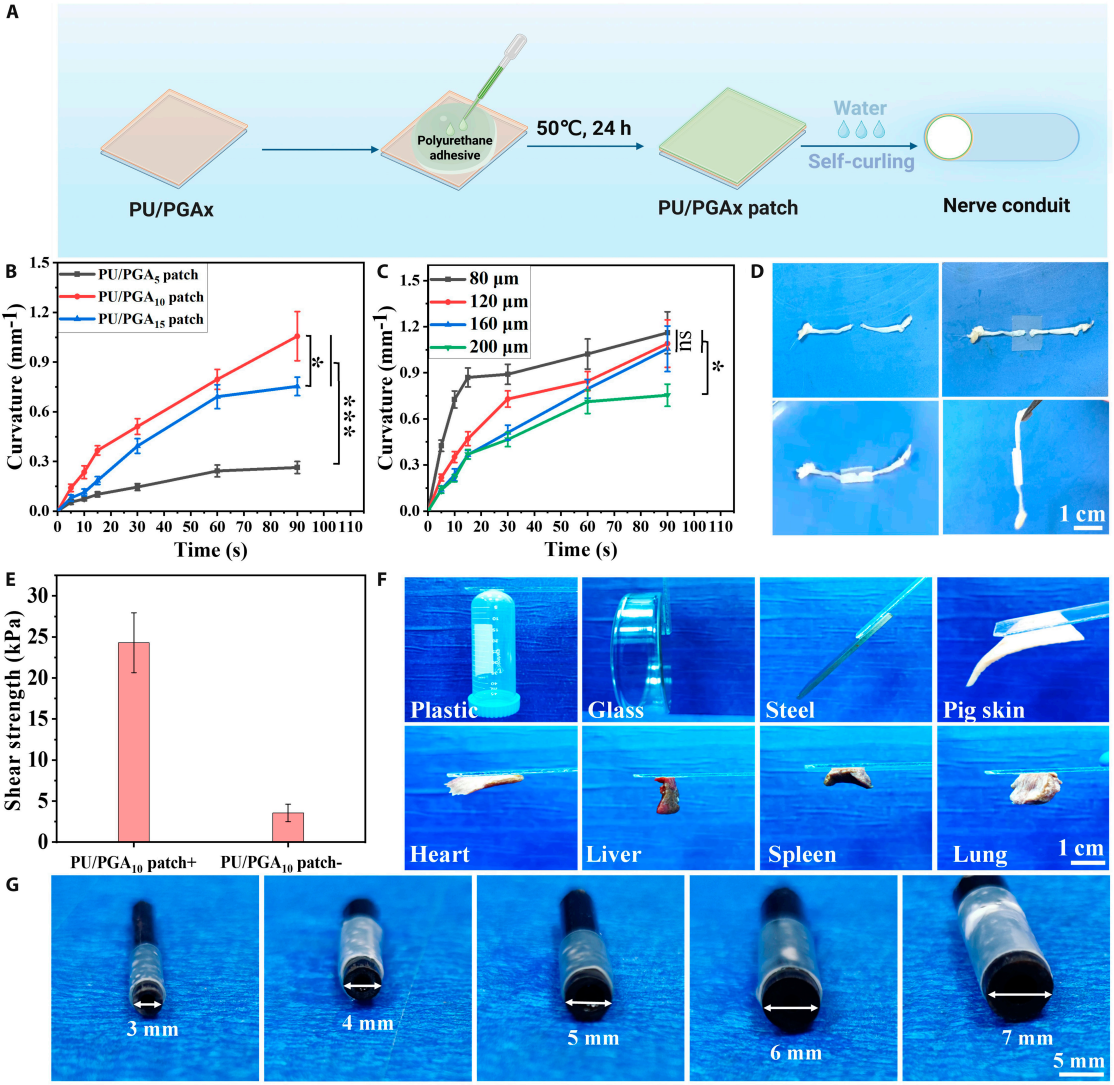
(A) Schematic diagram of the preparation of PU/PGA_*X*_ adhesive patches. (B) The curvature of PU/PGA_*X*_ patches changes with time after water immersion. (C) The curvature of PU/PGA_10_ patches with different thicknesses changes with time after water immersion. (D) The PU/PGA_10_ patch demonstrates conformable adhesive encapsulation of the rat sciatic nerve with adaptive wrapping in physiological saline. (E) The adhesion strength of the PU/PGA_10_ patch to pork skin. (F) PU/PGA_10_ patch adhered to different substrates. (G) The PU/PGA_10_ patch can be adapted to tubular models with different diameters.

The ideal nerve conduit scaffold should possess curling capability and adhesive properties to enable adaptive wrapping and secure fixation of nerve tissue. Our group has previously developed a series of waterborne PU emulsion adhesives for tissue adhesion and wound repair [24]. The PU adhesive emulsion was synthesized according to our previous literature report and coated onto the self-curling film for functional integration to fabricate the self-curling adhesive conduit (Fig. 3A). The bending curvature of the PU/PGA_*X*_ adhesive conduit after soaking in water for varying durations was calculated. As shown in Fig. 3B, after soaking in water for 90 s, the maximum bending curvatures of PU/PGA_5_, PU/PGA_10_, and PU/PGA_15_ adhesive films were 0.25, 1.02, and 0.77 mm^−1^, respectively. The PU/PGA_10_ patch demonstrates the fastest curling speed and the maximum bending curvature. This is likely because, with a PGA content of 10%, a well-defined differential swelling distribution forms between the top and bottom layers of the film. However, when the PGA content increases to 15%, excessive hydrophilicity may blur the hydrophilic–hydrophobic interface, thereby weakening the stress gradient in the graded structure. At the same time, excessively high PGA content could reduce the rigid constraint provided by the hydrophobic PCL phase. Consequently, both the curling speed and curvature of the PU/PGA_15_ patch are lower than those of PU/PGA_10_. In contrast, when the PGA content is 5%, insufficient hydrophilic components lead to a reduced swelling driving force, which is insufficient to generate adequate swelling-driven force for rapid shape transformation. These results indicated that the amount of PGA added affects the coil curvature and velocity of PU/PGA_*X*_ films. Fig. 3C shows the change in the curl curvature of PU/PGA_10_ adhesive conduits with different thicknesses as a function of time after soaking in water. When the thickness of the patch decreased from 200 to 80 μm after soaking for 90 s, the maximum curvature increased from 0.75 to 1.17 mm^−1^. This may be related to the rate of water penetration; when the film thickness is thinner, the rapid water penetration leading to gradient swelling will be completed in a shorter time, thus accelerating the deformation response.

The PU/PGA_10_ adhesive conduit exhibits the fastest curling rate and the greatest curling curvature, making it the selected material for subsequent nerve injury repair. Fig. 3D depicts the PU/PGA_10_ patch being used for water-responsive self-curling to adhere and connect severed nerve ends. Stimulated by physiological saline, the patch self-rolls into a tubular shape, encapsulating and adhering the 2 severed nerve stumps to connect them. The adhesive strength of the PU/PGA_10_ patch to porcine skin was evaluated using a lap-shear model. The top side of the patch achieved an adhesion strength of 24.3 kPa to porcine skin, while the bottom side, lacking the coated waterborne PU adhesive, exhibited a significantly lower adhesion strength of only 4.56 kPa (Fig. 3E). This asymmetric adhesion profile is also advantageous for the in vivo application of the PU/PGA_10_ patch, as it prevents the bottom side from adhering to other tissues. Fig. 3F shows that the PU/PGA_10_ patch can stably adhere to plastic, glass, iron sheets, and various porcine tissues. To validate the adaptability and practicality of the PU/PGA_10_ adhesive conduit for neural tissue, black plastic tubes were placed in water together with PU/PGA_10_ adhesive patches to observe the patch’s curling behavior and its adhesion and wrapping around the tube. As shown in Fig. 3G, after immersion in water, PU/PGA_10_ patches can self-curl and effectively adhere to and wrap around black tubes with diameters ranging from 3 to 10 mm. This demonstrates that the PU/PGA_10_ patch possesses the ability to adapt to conduits of different sizes, showing promise for its application in connecting damaged nerves of various sizes.

### In vitro cell experiment

**Fig. 4.**
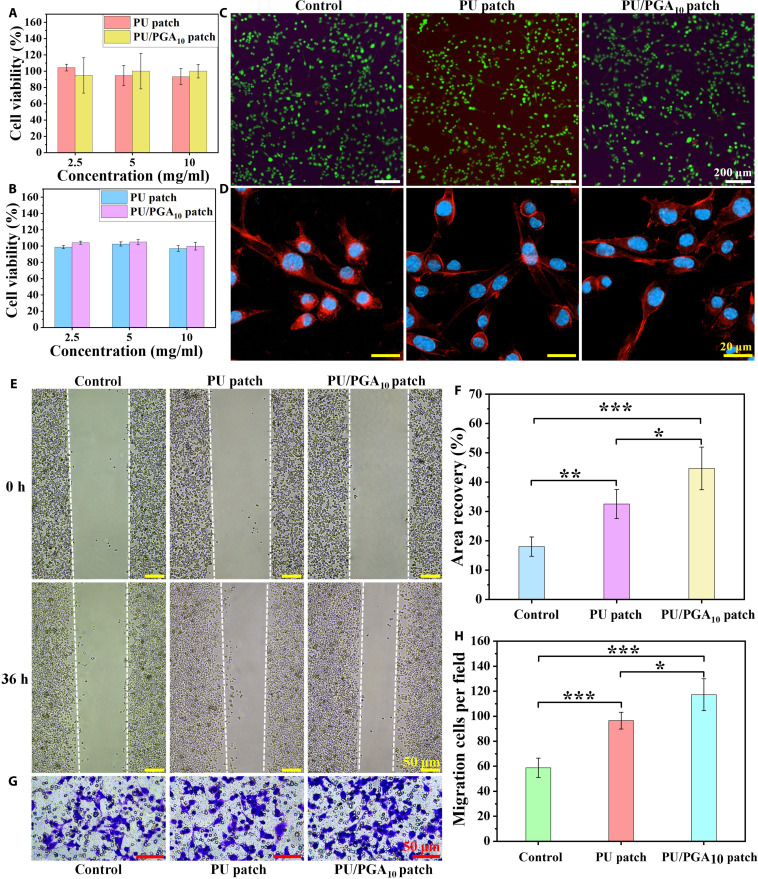
Cell viability of extracts of PU and PU/PGA_10_ patches at (A) 24 h and (B) 48 h. (C) Live/dead staining of rat Schwann cells (RSC96 cells) cultured with extracts of PU and PU/PGA_10_ patches for 48 h. (D) Cytoskeleton staining of RSC96 cells after coincubation with PU and PU/PGA_10_ patches for 24 h. (E) Cell scratch assay was used to test the effect of PU and PU/PGA_10_ patches on cell migration. (F) The recovery percentage of cell migration area in the scratch test. (G) The transwell assay was used to detect the effect of PU and PU/PGA_10_ patches on cell migration. (H) Statistics of cell migration in the field of the transwell experiment. Results are expressed as mean ± SD (*n* = 4).

The PU/PGA_10_ patch holds promise for application in sciatic nerve repair, and its biocompatibility is crucial for potential in vivo use. The cytocompatibility of both PU and PU/PGA_10_ patches was evaluated using the CCK-8 assay and live/dead staining. After coculturing RSC96 cells with extracts from the patches for 24 and 48 h, the results indicated that neither the PU nor the PU/PGA_10_ patch exhibited cytotoxicity; cell viability remained above 80% in both cases (Fig. 4A and B). At 48 h, RSC96 cells in both the PU and PU/PGA_10_ patch groups displayed extensive green fluorescence, comparable to the control group (Fig. 4C). Furthermore, RSC96 cells were cocultured with PU and PU/PGA_10_ patches for 24 h and then subjected to cytoskeleton staining. As shown in Fig. 4D, compared to the blank control group, RSC96 cells treated with PU or PU/PGA_10_ patches showed no obvious changes in their nuclei, and cellular activity remained normal. These results demonstrate that the PU/PGA_10_ patch exhibits favorable cytocompatibility, suggesting its potential for repairing nerve injuries in vivo.

RSC96 cells were cocultured with extracts from the PU patch and PU/PGA_10_ patch to investigate the effects of these materials on cell migration. After 48 h of culture, compared to the control group, the PU/PGA_10_ patch exhibited the strongest ability to promote cell migration (Fig. 4E and F). This finding was further confirmed by Transwell migration assays, which demonstrated that the PU/PGA_10_ patch group induced a more significant enhancement in cell migration compared to both the PU patch group and the control group (Fig. 4G and H). These results indicate that both PU and PU/PGA_10_ patches, notably the PU/PGA_10_ patch, significantly enhance cellular migration capacity, thereby providing a favorable microenvironment for cell spreading and growth. The improved cell spreading and migration observed with the PU/PGA_10_ patch may be associated with the incorporation of PGA. PGA likely enhances cell–material interactions, leading to better adhesion, spreading, and migration of RSC96 cells [37,38].

An in vitro macrophage model was established using RAW264.7 cells to simulate the immune microenvironment. RAW264.7 cells were seeded onto culture plates preloaded with or without patches, followed by stimulation with lipopolysaccharide (LPS). After 8 h of LPS stimulation, the expression of tumor necrosis factor-α (TNF-α) was reduced in the PU/PGA_10_ patch group compared with the control group and the PU patch group (Fig. S6A). Conversely, the mRNA expression of the M2-related marker interleukin-10 was higher in the PU/PGA_10_ patch group than in the other 2 groups (Fig. S6B). Culture supernatants were collected after 24 h of LPS stimulation and analyzed by enzyme-linked immunosorbent assay. Compared with the control group and the PU patch group, the level of TNF-α was lower in the PU/PGA_10_ patch group (Fig. S6C and D). These findings suggest that the PU/PGA_10_ patch possesses certain immunomodulatory effects, capable of suppressing inflammatory responses and promoting the transformation of macrophages toward a phenotype more conducive to repair [39–41].

### The subcutaneous implantation and degradation performance of adhesive conduit

To further validate the in vivo biosafety of the adhesive patch, the PU/PGA_10_ patch was subcutaneously implanted in rats, and the implantation condition was observed via hematoxylin and eosin (H&E) staining images. As shown in Fig. S7A and B, 2 weeks after implantation, a thin layer of fibrous capsule was observed encapsulating the patch material, with no obvious tissue inflammatory response noted. The degradation behavior of the PU/PGA_10_ patch in vitro was observed using a lipase solution. As shown in Fig. S8A, the mass loss of the PU/PGA_10_ patch was 31.4% at week 2 and reached 51.1% by week 20. The patch degraded relatively rapidly during the initial stage, likely due to the dissolution, release, or degradation of PGA and components in the amorphous regions. The in vivo degradation behavior of the PU/PGA_10_ patch was assessed via subcutaneous implantation in rats. As shown in Fig. S8B, the patch was gradually degraded and absorbed subcutaneously over time, with most of it being degraded by week 20.

### Adhesive conduit for rat sciatic nerve repair

**Fig. 5.**
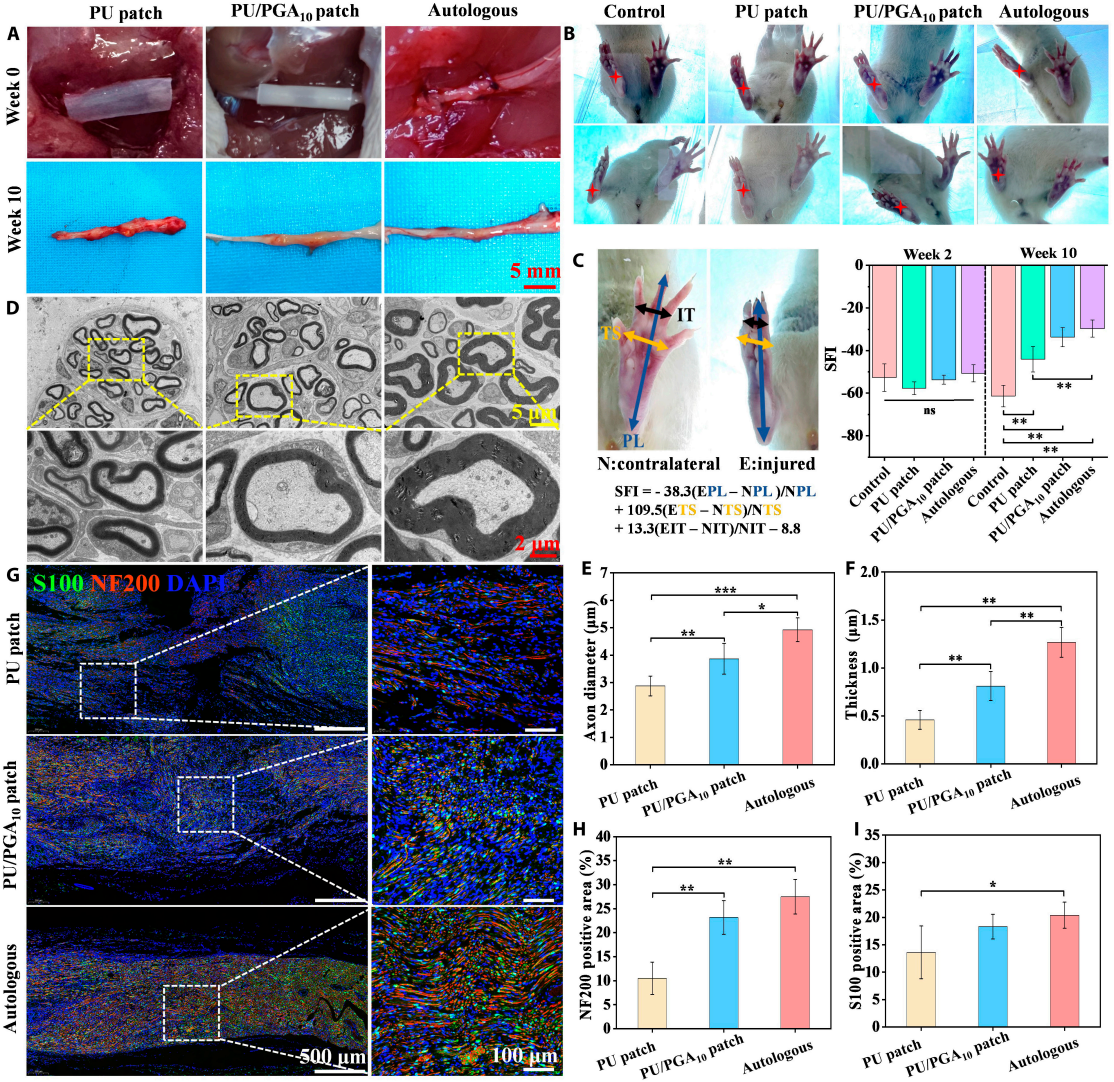
(A) Surgical pictures of the PU patch, PU/PGA_10_ patch, and autologous for the treatment of sciatic nerve injury in rats. (B) Footprint photos of mice in each group at 2 and 10 weeks after surgery, with asterisks indicating injured limbs. (C) Sciatic function index (SFI) calculation method and SFI values of each group. (D) Transmission electron microscopy (TEM) of the optimal myelination index of the regenerated nerve at 10 weeks after surgery. (E) Axon diameter and (F) myelin thickness of the regenerated nerve at 10 weeks after surgery. (G) Immunofluorescence staining and (H and I) positive area percentage of NF200 and S100 in the regenerated nerve of each group at 10 weeks after operation. Results are expressed as mean ± SD (*n* = 4). *P* value less than 0.05 was considered significant, *P < 0.05, **P < 0.01, ***P < 0.001.

The PU/PGA_10_ patch demonstrates water-responsive self-curling capability, adhesiveness, and biocompatibility in vitro, suggesting its potential for reconnecting damaged nerves to facilitate repair. The ability of the PU/PGA_10_ patch to induce nerve regeneration was evaluated using the rat sciatic nerve long segment (8 mm) injury model, with autologous nerve transplantation serving as the positive control group. As shown in Fig. 5A and Movie S2, the PU/PGA_10_ patch successfully reconnected the 2 severed nerve stumps through its self-curling encapsulation and adhesive functions. In contrast, the PU patch (without PGA) required manual wrapping and attachment to the nerve stumps to achieve reconnection. Rats were sacrificed 10 weeks postoperation, and the conduits were removed to assess the repair outcomes. As shown in Fig. 5A, obvious new tissue formation was observed in the PU patch, PU/PGA_10_ patch, and autograft groups. Moreover, the PU/PGA_10_ patch and autograft groups exhibited complete and robust regenerated nerves, and the repair effects were better than those of the PU patch group.

Sciatic nerve injury impairs motor function in rats, leading to abnormal footprints. By 10 weeks postoperation, footprint analysis revealed severe curling of the plantar surface of the ipsilateral hind paw in the untreated control group, preventing normal extension. In contrast, treatment groups exhibited partial toe extension (Fig. 5B). The recovery of motor function in each treatment group was evaluated using the SFI scoring system. Normal rats exhibit an SFI value of 0, which decreases with increasing injury severity, reaching −100 for complete transection [32]. As shown in Fig. 5C, at 2 weeks postoperation, the SFI values for the PU patch, PU/PGA_10_ patch, and autograft groups were −57.34, −53.29, and −50.74, respectively. These values showed no significant difference compared to the untreated control group (−52.61), indicating that motor function had not yet recovered in any group at this early time point. By 10 weeks postoperation, the SFI values for the PU patch, PU/PGA_10_ patch, and autograft groups were −44.04, −33.68, and −29.66, respectively, all significantly higher than that of the untreated control group (−61.37). These findings indicate partial recovery of motor function in all treated groups, with the PU/PGA_10_ patch and autograft groups demonstrating superior repair efficacy compared to the PU patch group.

The growth of axons and myelin sheaths in regenerated nerves was examined using TEM. As revealed in Fig. 5D, regenerated axons in the PU patch group were unevenly distributed and relatively sparse. In contrast, uniformly distributed axons with myelin sheath encapsulation were observed in both the PU/PGA_10_ patch group and the autograft group. At 10 weeks postoperation, the average axon diameters in the PU patch, PU/PGA_10_ patch, and autograft groups were 2.80, 3.92, and 4.85 μm, respectively (Fig. 5E). The increased axon diameters observed in the PU/PGA_10_ patch and autograft groups can significantly enhance nerve impulse conduction velocity. Similarly, the average myelin sheath thicknesses were 0.46, 0.81, and 1.29 μm for the PU patch, PU/PGA_10_ patch, and autograft groups, respectively (Fig. 5F). The increased myelin sheath thickness in the PU/PGA_10_ patch and autograft groups indicates a higher degree of myelination and superior quality of nerve regeneration. The calculated optimal myelin formation index (*g*-ratio) values for the PU patch, PU/PGA_10_ patch, and autograft groups were 0.68, 0.63, and 0.56, respectively (Fig. S9). Lower *g*-ratio values signify higher-quality regenerated nerves. Larger axon diameters combined with thicker myelin sheaths improve nerve conduction velocity and efficiency, and are closely correlated with enhanced recovery of motor function [42].

**Fig. 6.**
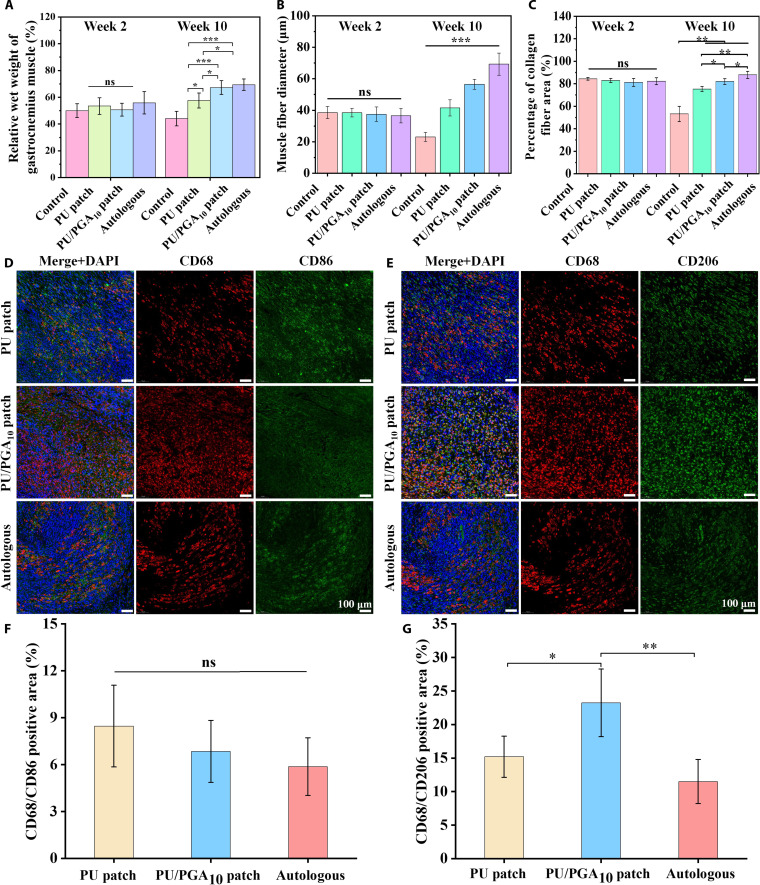
(A) Wet weight ratio of gastrocnemius muscle of operated and healthy sides in each group. Statistical analysis of (B) muscle fiber diameter and (C) muscle fiber area percentage in the Masson staining images of the gastrocnemius muscle in each group at 2 weeks and 10 weeks after surgery. (D and E) Immunofluorescence staining of macrophage polarization phenotype and (F and G) percentage of colocalized positive cells at 2 weeks after surgery. Results are expressed as mean ± SD (*n* = 4).

To further evaluate peripheral nerve regeneration and repair in the treated rats, the histological morphology of the regenerated nerves was examined using immunofluorescence staining. The regenerated nerves were immunostained with markers for axons (neurofilament 200, NF200), Schwann cells (S100), and nuclei (4′,6-diamidino-2-phenylindole) [43]. As shown in Fig. 5G, at 10 weeks postoperation, robust axonal regeneration bridging the proximal and distal stumps was observed in both the autograft group and the PU/PGA_10_ patch group. In contrast, the PU patch group exhibited incomplete nerve reconnection, with an evident gap at the bridging site. Furthermore, widely distributed regenerated axons and significantly greater density of NF200 and S100 positive expression were observed in the autograft and PU/PGA_10_ patch groups compared to the PU patch group (Fig. 5H and I). The vascularization in the regenerated nerves of each group was evaluated by immunofluorescence staining for CD31 and alpha-smooth muscle actin (α-SMA). As shown in Fig. S10A and B, compared with the PU patch group, the colocalized positive expression of CD31 and α-SMA was higher in the autograft group and the PU/PGA_10_ patch group. The blood vessels in the regenerated nerves provide essential nutrients and oxygen for nerve regeneration, indicating better repair effects in the autograft group and the PU/PGA_10_ patch group. At 10 weeks postoperation, the gastrocnemius muscles from both the operated (ipsilateral) and contralateral normal sides were harvested. The degree of muscle atrophy was quantitatively assessed by calculating the wet weight ratio of the operated-side muscle relative to the contralateral normal-side muscle. As shown in Fig. 6A, the wet weight ratios for the PU patch, PU/PGA_10_ patch, and autograft groups at 10 weeks were 57.57%, 67.26%, and 69.39%, respectively. All treatment groups exhibited significantly higher ratios compared to the untreated control group (44.06%). These results demonstrate that both the conduit scaffolds (PU/PGA_10_ patch) and autografts promoted nerve regeneration, effectively maintaining cellular water retention in muscle tissue and reducing atrophic degeneration. The histological morphology of the gastrocnemius muscle was observed through Masson staining, and the diameters of muscle fibers in each group were statistically analyzed. Fig. S11 and Fig. 6B showed that the average diameters of muscle fibers in the PU patch, PU/PGA_10_ patch, and autologous transplantation groups at 10 weeks after surgery were 41.51, 56.52, and 69.28 μm, respectively, which were significantly higher than that of the control group (23.03 μm). This result indicates that the muscle fiber structure in each treatment group has improved, the degree of atrophy has decreased, and the therapeutic effects were more obvious in the PU/PGA_10_ patch and autologous transplantation groups. Additionally, the area percentage of muscle fibers in the control group decreased from 84.37% at 2 weeks to 53.18% at 10 weeks (Fig. 6C), indicating severe muscle fibrosis. These results suggest that the treatment intervention with patch catheters and autologous transplantation can inhibit fibroblast activation and excessive deposition of ECM, avoiding muscle atrophy [44].

The comparative analysis of motor functional recovery, histological nerve regeneration, ultrastructural observations, and gastrocnemius muscle atrophy collectively demonstrates that the PU/PGA_10_ patch facilitated superior nerve regeneration and repair compared to the PU patch alone. To investigate the inflammatory response, the number and activation state of macrophages at the nerve coaptation site were assessed via immunofluorescence staining at 2 weeks postoperation. CD68 served as a general macrophage marker, CD86 expression indicated the activation level of pro-inflammatory M1 macrophages, and CD206 marked anti-inflammatory M2 macrophages associated with tissue repair [45]. As shown in Fig. 6D and E, the positive expression of M1 phenotype macrophages (CD86^+^) showed no significant difference among groups at 2 weeks postoperation. However, the PU/PGA10 patch group exhibited significantly higher positive expression of M2 phenotype macrophages (CD206^+^) compared to the PU patch group (Fig. 6F and G). This finding suggests that the incorporation of PGA promotes macrophage polarization toward the M2 reparative phenotype. M2 macrophages optimize the regenerative microenvironment by not only secreting anti-inflammatory cytokines to suppress local inflammation but also releasing neurotrophic factors and pro-angiogenic factors, thereby enhancing nerve regeneration [46].

Since PU patches lack a self-curling response function and must be manually wrapped and adhered to bridge the 2 nerve stumps, their adaptability for nerve wrapping is inferior to that of PU/PGA_10_ patches. Although the PU/PGA_10_ patch also requires positioning operations in the initial stage, this process is faster and more straightforward compared to the PU patch. Therefore, the repair mechanism of PU/PGA_10_ patches in peripheral nerve regeneration is primarily attributed to their ability to provide an oriented growth microenvironment for neural regeneration through adaptive fixation and bridging of the injured nerve. Additionally, PGA-incorporated PU/PGA_10_ patches can further enhance nerve regeneration by promoting macrophage polarization toward the M2 reparative phenotype. Moreover, this adhesive patch with self-curling and adhesive characteristics may also be applied in spatially confined or hard-to-suture areas for tissue repair. Regarding the water-responsive self-curling PU/PGA_*X*_ patch, the current control over its curling dynamics and curvature remains insufficiently precise. Models already exist for the bending deformation behavior of bilayer structures composed of different materials under external stimuli, such as the Timoshenko bilayer model, which can predict bending deformation behavior based on the properties of the bilayer materials [47,48]. Future research could employ a combined approach of computer simulations and experimental studies to quantitatively elucidate the influence of the distribution parameters of hydrophilic/hydrophobic components between membrane layers on the curling dynamics and curvature. This would facilitate the establishment of a structure-property modulation model to guide the development of customized self-curling patches. Currently, there have also been reports on adhesives for nerve repair. However, most of them merely connect or fix the severed nerve ends using adhesive glues or hydrogels [49]. Meanwhile, self-adhesive patches or conduits often require techniques such as immediate in situ formation, laser stimulation, and 3D printing, which can cause inconvenience during surgical procedures [50–52]. In contrast, the PU/PGA_10_ patch features self-curling adhesion and fixation, offering greater convenience in both preparation and operation. This makes it a promising candidate for applications in the field of nerve repair.

## Conclusion

In summary, we have developed an adhesive conduit possessing water-responsive self-curling and adhesive capabilities for peripheral nerve repair. Upon drying a mixture of PU emulsion and PGA solution, the resulting film forms with the hydrophobic PU enriched at the top surface and the hydrophilic PGA predominantly at the bottom. The differential swelling of these hydrophilic and hydrophobic components upon water exposure drives the film to autonomously curl into a closed tubular structure, mimicking the movement of pinecone scales. Functional integration was achieved by coating the self-curling film with a PU adhesive emulsion, yielding a self-curling adhesive patch. This patch can adaptively wrap around, adhere to, and stably fixate nerve tissue under aqueous stimulation, eliminating the need for sutures. This approach effectively prevents secondary iatrogenic damage to the injured nerve and offers straightforward application. In rat models of PNI, the PU/PGA patch robustly promoted the polarization of macrophages toward the pro-regenerative M2 phenotype. By 10 weeks postoperation, it enhanced nerve regeneration and functional recovery. The development of this self-curling adhesive conduit holds new promise for the clinical translation of nerve repair strategies.

## Data Availability

The data are contained within the article and the Supplementary Materials.

## Supplementary Materials

Supplementary 1 Characterization of adhesive patches Coarse-grained molecular dynamics simulation Macrophage polarization in vitro The degradation performance of the adhesive patch Figs. S1 to S11 Tables S1 to S4 Movies S1 and S2

## References

[1] Tansey MG, Wallings RL, Houser MC, Herrick MK, Keating CE, Joers V. Inflammation and immune dysfunction in Parkinson disease. Nat Rev Immunol. 2022;22(11):657–673. 35246670 10.1038/s41577-022-00684-6 PMC8895080

[2] Kim S, Oh YS, Lee K, Kim S, Maeng W-Y, Kim KS, Kim G-B, Cho S, Han H, Park H, et al. Battery-free, wireless, cuff-type, multimodal physical sensor for continuous temperature and strain monitoring of nerve. Small. 2023;19(32): Article e2206839. 37069777 10.1002/smll.202206839

[3] Li M, Tang Y, Zhou C, Geng Y, Zhang C, Hsu Y, Ma L, Guo W, Li M, Wang Y. The application of stem cells and exosomes in promoting nerve conduits for peripheral nerve repair. Biomater Res. 2025;29:0160. 40231207 10.34133/bmr.0160 PMC11994886

[4] Liu Y, Xu YJ. LKB1 and CRMP1 cooperatively promote the repair of the sciatic nerve injury. Dev Neurobiol. 2023;84(1):18–31. 38105470 10.1002/dneu.22932

[5] Li J, Xie Y, Zhao J, Zhao F, Bahatibieke A, Liu G, Meng H, Zheng Y. A self-powered microsphere-electret/conductive bacterial cellulose composite for sciatic nerve repair. Compos Part B Eng. 2025;297:112246.

[6] Wei S, Hu Q, Ma J, Dai X, Sun Y, Han G, Meng H, Xu W, Zhang L, Ma X, et al. Acellular nerve xenografts based on supercritical extraction technology for repairing long-distance sciatic nerve defects in rats. Bioact Mater. 2022;18:300–320. 35387172 10.1016/j.bioactmat.2022.03.014 PMC8961471

[7] Qi T, Zhang X, Gu X, Cui S. Experimental study on repairing peripheral nerve defects with novel bionic tissue engineering. Adv Healthc Mater. 12(17):e2203199. 10.1002/adhm.202203199 PMC11469147 36871174

[8] Luo L, He Y, Jin L, Zhang Y, Guastaldi FP, Albashari AA, Hu F, Wang X, Wang L, Xiao J, et al. Application of bioactive hydrogels combined with dental pulp stem cells for the repair of large gap peripheral nerve injuries. Bioact Mater. 2021;6(3):638–654. 33005828 10.1016/j.bioactmat.2020.08.028 PMC7509005

[9] Li C, Guo C, Fitzpatrick V, Ibrahim A, Zwierstra MJ, Hanna P, Lechtig A, Nazarian A, Lin SJ, Kaplan DL. Design of biodegradable, implantable devices towards clinical translation. Nat Rev Mater. 2019;5(1):61–81.

[10] Zhang C, Gong J, Zhang J, Zhu Z, Qian Y, Lu K, Zhou S, Gu T, Wang H, He Y, et al. Three potential elements of developing nerve guidance conduit for peripheral nerve regeneration. Adv Funct Mater. 2023;33(40):2302251.

[11] Cai Y, Chen Y, Zhang G, Lin Y, Zhang J, Liang J, Lv L, Wang Y, Fang X, Dang X. The GDNF-gel/HA-Mg conduit promotes the repair of peripheral nerve defects by regulating PPAR-γ/RhoA/ROCK signaling pathway. iScience. 2024;27(2):108969. 38322994 10.1016/j.isci.2024.108969 PMC10844047

[12] Li X, Mao X, Tao M, Liang F, Tian X, Fan J, Wang X, Yu T, Ao Q. Enhancing neuroinduction activity of PLCL-based nerve conduits through native epineurium integration. Biomater Adv. 2024;159:213803. 38447384 10.1016/j.bioadv.2024.213803

[13] Wang J, Cheng Y, Wang H, Wang Y, Zhang K, Fan C, Wang H, Mo X. Biomimetic and hierarchical nerve conduits from multifunctional nanofibers for guided peripheral nerve regeneration. Acta Biomater. 2020;117:180–191. 33007489 10.1016/j.actbio.2020.09.037

[14] Wu W, Dong Y, Liu H, Jiang X, Yang L, Luo J, Hu Y, Gou M. 3D printed elastic hydrogel conduits with 7,8-dihydroxyflavone release for peripheral nerve repair. Mater Today Bio. 2023;20:100652. 10.1016/j.mtbio.2023.100652 PMC10199216 37214548

[15] Escobar A, Serafin A, Carvalho MR, Culebras M, Cantarero A, Beaucamp A, Reis RL, Oliveira JM, Collins MN. Electroconductive poly (3,4-ethylenedioxythiophene) (PEDOT) nanoparticle-loaded silk fibroin biocomposite conduits for peripheral nerve regeneration. Adv Compos Hybrid Mater. 2023;6(3):118.

[16] Song J, Dong J, Yuan Z, Huang M, Yu X, Zhao Y, Shen Y, Wu J, El-Newehy M, Abdulhameed MM. Shape-persistent conductive nerve guidance conduits for peripheral nerve regeneration. Adv Healthc Mater. 2024;13(26):et al., Article e2401160. 10.1002/adhm.202401160 38757919

[17] Wang Q, Wei Y, Yin X, Zhan G, Cao X, Gao H. Engineered PVDF/PLCL/PEDOT dual electroactive nerve conduit to mediate peripheral nerve regeneration by modulating the immune microenvironment. Adv Funct Mater. 2024;34(28):2400217.

[18] Wan T, Li QC, Zhang FS, Zhang XM, Han N, Zhang PX. Biomimetic ECM nerve guidance conduit with dynamic 3D interconnected porous network and sustained IGF-1 delivery for enhanced peripheral nerve regeneration and immune modulation. Mater Today Bio. 2025;30:101403. 10.1016/j.mtbio.2024.101403 PMC11713512 39790488

[19] Zhang M, An H, Gu Z, Zhang YC, Wan T, Jiang HR, Zhang FS, Jiang BG, Han N, Wen Y-Q, et al. Multifunctional wet-adhesive chitosan/acrylic conduit for sutureless repair of peripheral nerve injuries. Int J Biol Macromol. 2023;253(Pt 6):126793. 37709238 10.1016/j.ijbiomac.2023.126793

[20] Yang Z, Wang Y, Lan L, Wang Y, Zhang X. Bioinspired H-bonding connected gradient nanostructure actuators based on cellulose nanofibrils and graphene. Small. 2024;20(36): Article e2401580. 38708893 10.1002/smll.202401580

[21] Mariani S, Cecchini L, Mondini A, Del Dottore E, Ronzan M, Filippeschi C, Pugno NM, Sinibaldi E, Mazzolai B. A bioinspired plasmonic nanocomposite actuator sunlight-driven by a photothermal-hygroscopic effect for sustainable soft robotics. Adv Mater Technol. 2023;8(14):2202166.

[22] Kim K, Guo Y, Bae J, Choi S, Song HY, Park S, Hyun K, Ahn SK. 4D printing of hygroscopic liquid crystal elastomer actuators. Small. 17(23): Article e2100910. 10.1002/smll.202100910 33938152

[23] Wei S, Ghosh TK. Bioinspired structures for soft actuators. Adv Mater Technol. 2022;7(10):2101521.

[24] Guo X, Wang A, Sheng N, He Y, Liu W, Li Z, Luo F, Li J, Tan H. Janus polyurethane adhesive patch with antibacterial properties for wound healing. ACS Appl Mater Interfaces. 2024;16(13):15970–15980. 38501704 10.1021/acsami.4c00924

[25] Zhang D, Liu J, Chen B, Zhao Y, Wang J, Ikeda T, Jiang L. A hydrophilic/hydrophobic Janus inverse-opal actuator via gradient infiltration. ACS Nano. 2018;12(12):12149–12158. 30418739 10.1021/acsnano.8b05758

[26] Lv C, Xia H, Shi Q, Wang G, Wang YS, Chen QD, Zhang YL, Liu LQ, Sun HB. Sensitively humidity-driven actuator based on photopolymerizable PEG-DA films. Adv Mater Interfaces. 2017;4(9):1601002.

[27] Zhang Y, He W, Li J, Wang K, Li J, Tan H, Fu Q. Gemini quaternary ammonium salt waterborne biodegradable polyurethanes with antibacterial and biocompatible properties. Mater Chem Front. 2017;1(2):361–368.

[28] He W, Zhang Y, Li J, Gao Y, Luo F, Tan H, Wang K, Fu Q. A novel surface structure consisting of contact-active antibacterial upper-layer and antifouling sub-layer derived from gemini quaternary ammonium salt polyurethanes. Sci Rep. 2016;6:32140. 27561546 10.1038/srep32140 PMC4999876

[29] Xu P, Wang Y, Cheng P, Cong P, Li D, Zhang Z, Hui J, Ye M. Toughness modification of waterborne epoxy emulsified asphalt by waterborne polyurethane elastomer. Constr Build Mater. 2023;386:131547.

[30] Liu B, Che C, Liu J, Si M, Gong Z, Li Y, Zhang J, Yang G. Fabrication and antitumor mechanism of a nanoparticle drug delivery system: Graphene oxide/chitosan oligosaccharide/γ-polyglutamic acid composites for anticancer drug delivery. ChemistrySelect. 2019;4(43):12491–12502.

[31] Pisani S, Dorati R, Scocozza F, Mariotti C, Chiesa E, Bruni G, Genta I, Auricchio F, Conti M, Conti B. Preliminary investigation on a new natural based poly(gamma-glutamic acid)/chitosan bioink. J Biomed Mater Res Part B Appl Biomater. 2020;108(7):2718–2732. 10.1002/jbm.b.34602 32159925

[32] Wang R, Wang X, Zhan Y, Xu Z, Xu Z, Feng X, Li S, Xu H. A dual network hydrogel sunscreen based on poly-γ-glutamic acid/tannic acid demonstrates excellent anti-UV, self-recovery, and skin-integration capacities. ACS Appl Mater Interfaces. 2019;11(41):37502–37512. 31544451 10.1021/acsami.9b14538

[33] Tehrani AG, Makki H, Anbaran SRG, Vakili H, Ghermezcheshme H, Zandi N. Superior anti-biofouling properties of mPEG-modified polyurethane networks via incorporation of a hydrophobic dangling chain. Prog Org Coat. 2021;158:106358.

[34] Galhenage TP, Webster DC, Moreira AMS, Burgett RJ, Stafslien SJ, Vanderwal L, Finlay JA, Franco SC, Clare AS. Poly(ethylene) glycol-modified, amphiphilic, siloxane–polyurethane coatings and their performance as fouling-release surfaces. J Coat Technol Res. 2016;14(2):307–322.

[35] Zhang Y, He X, Ding M, He W, Li J, Li J, Tan H. Antibacterial and biocompatible cross-linked waterborne polyurethanes containing gemini quaternary ammonium salts. Biomacromolecules. 2018;19(2):279–287. 29253335 10.1021/acs.biomac.7b01016

[36] Zhou R, Jin Y, Zeng W, Jin H, Li Y, Mei J, Liu J. Janus hydrophobic structural gel with asymmetric adhesion in air/underwater for reliable mechanosensing. Adv Funct Mater. 2024;34(33):2316687.

[37] Uddin Z, Fang TY, Siao JY, Tseng WC. Wound healing attributes of polyelectrolyte multilayers prepared with multi-l-arginyl-poly-l-aspartate pairing with hyaluronic acid and γ-polyglutamic acid. Macromol Biosci. 2020;20(8): Article e2000132. 32567226 10.1002/mabi.202000132

[38] Yang R, Huang J, Zhang W, Xue W, Jiang Y, Li S, Wu X, Xu H, Ren J, Chi B. Mechanoadaptive injectable hydrogel based on poly(γ-glutamic acid) and hyaluronic acid regulates fibroblast migration for wound healing. Carbohydr Polym. 2021;273:118607. 34561006 10.1016/j.carbpol.2021.118607

[39] Lima BV, Oliveira MJ, Barbosa MA, Gonçalves RM, Castro F. Harnessing chitosan and poly-(γ-glutamic acid)-based biomaterials towards cancer immunotherapy. Mater Today Adv. 2022;15:100252.

[40] Ahn H, Kang SG, Yoon SI, Kim PH, Kim D, Lee GS. Poly-gamma-glutamic acid from *Bacillus* subtilis upregulates pro-inflammatory cytokines while inhibiting NLRP3, NLRC4 and AIM2 inflammasome activation. Cell Mol Immunol. 2016;15(2):111–119. 27133472 10.1038/cmi.2016.13 PMC5811673

[41] Li J, Huang Y, Wang Y, Han Q. A poly-γ-glutamic acid/ε-polylysine hydrogel: Synthesis, characterization, and its role in accelerated wound healing. Gels. 2025;11(4):226. 40277663 10.3390/gels11040226 PMC12027117

[42] Yan L, Liu S, Wang J, Ding X, Zhao Y, Gao N, Xia Z, Li M, Wei Q, Okoro OV, et al. Constructing nerve guidance conduit using dECM-doped conductive hydrogel to promote peripheral nerve regeneration. Adv Funct Mater. 2024;34(38):2402698.

[43] Song J, Wu S, Liao C, Yuan Z, Yu X, Shang P, Shen Y, Cui J, Wu J, Sun B, et al. Spiral-structured electrospun conductive conduits filled with aligned nanofibers for peripheral nerve regeneration. Chem Eng J. 2025;508:160899.

[44] Zhang S, Wang J, Zheng Z, Yan J, Zhang L, Li Y, Zhang J, Li G, Wang X, Kaplan D. Porous nerve guidance conduits reinforced with braided composite structures of silk/magnesium filaments for peripheral nerve repair. Acta Biomater. 2021;134:116–130. 34289421 10.1016/j.actbio.2021.07.028

[45] Wang Y, Lin J, Chen J, Liang R, Zhang Q, Li J, Shi M, Li L, He X, Lan T, et al. Biodegradable polyurethane-incorporating decellularized spinal cord matrix scaffolds enhance Schwann cell reprogramming to promote peripheral nerve repair. J Mater Chem B. 2023;11(10):2115–2128. 36779440 10.1039/d2tb02679a

[46] Zhu Q, Hong Y, Huang Y, Zhang Y, Xie C, Liang R, Li C, Zhang T, Wu H, Ye J, et al. Polyglutamic acid-based elastic and tough adhesive patch promotes tissue regeneration through in situ macrophage modulation. Adv Sci. 2022;9(17): Article e2106115. 10.1002/advs.202106115 PMC9189670 35396785

[47] Zhang Y, Ionov L. Controllable self-rolling of polyurethane/SiO_2_ film with differential density. Eur Polym J. 2019;119:32–36.

[48] Chen Y, Liao X, Zhao W, Yang P, Xiao H, Liu Y, Chen X. Post-wrinkling behaviors of a bilayer on a soft substrate. Int J Solids Struct. 2021;214–215:74–79.

[49] Liang X, Cai H, Hao Y, Sun G, Song Y, Chen W. Sciatic nerve repair using adhesive bonding and a modified conduit. Neural Regen Res. 2014;9(6):594–601. 25206861 10.4103/1673-5374.130099 PMC4146232

[50] Xue W, Shi W, Kuss M, Kong Y, Alimi OA, Wang HJ, DiMaio DJ, Yu C, Duan B. A dual-network nerve adhesive with enhanced adhesion strength promotes transected peripheral nerve repair. Adv Funct Mater. 2022;33(2):2209971. 36816838 10.1002/adfm.202209971 PMC9937437

[51] Sliow A, Ma Z, Gargiulo G, Mahns D, Mawad D, Breen P, Stoodley M, Houang J, Kuchel R, Tettamanzi GC, et al. Stimulation and repair of peripheral nerves using bioadhesive graft-antenna. Adv Sci. 2019;6(11):1801212. 10.1002/advs.201801212 PMC6548953 31179205

[52] Zhang J, Chen Y, Huang Y, Wu W, Deng X, Liu H, Li R, Tao J, Li X, Liu X, et al. A 3D-printed self-adhesive bandage with drug release for peripheral nerve repair. Adv Sci. 2020;7(23):2002601. 10.1002/advs.202002601 PMC7709979 33304766

