# Supplementary material for: Pinecone-Inspired Water-Responsive Curling Adhesive Conduit for Peripheral Nerve Repair
Source: Cyborg Bionic Syst. 2026 Mar 27;7:0556. doi: 10.34133/cbsystems.0556 (PMC13022317; doi:10.34133/cbsystems.0556)
Supplement: Supplementary 1 — Characterization of adhesive patches Coarse-grained molecular dynamics simulation Macrophage polarization in vitro The degradation performance of the adhesive patch Figs. S1 to S11 Tables S1 to S4 Movies S1 and S2 [file cbsystems.0556.f1.zip › Supplementary Materials.docx]

**Pinecone-Inspired Water-Responsive Curling Adhesive Conduit for Peripheral Nerve Repair**

Xiaolei Guo^a,1^, Jinwei Li^c,1^, Hongyu Xu^d^, Shengrong Long^d,e^, Junhong Li^c^, Ao Wang^a^, Wenkai Liu^a^, Fan Zhang^c^, Zhen Li^a^, Feng Luo^a^, Jiehua Li^a^, Yanchao Wang^a,c,*^, Hong Tan^a,*^, Ting Lan^b,*^

^a^ College of Polymer Science and Engineering, National Key Laboratory of Advanced Polymer Materials, Med-X Center for Materials, Sichuan University, Chengdu 610065, China

^b^ Department of Pathology, Sichuan Clinical Research Center for Cancer, Sichuan Cancer Hospital & Institute, Sichuan Cancer Center, University of Electronic Science and Technology of China, Chengdu, 610000, China.

^c^ Department of Neurosurgery, West China Hospital, Sichuan University, Chengdu, Sichuan, 610000, China.

^d^ Brain Research Center, Zhongnan Hospital of Wuhan University, Wuhan University, Wuhan, 430071, China.

^e^ Department of Thoracic Surgery, Tongji Hospital, Tongji Medical College, Huazhong University of Science and Technology, Wuhan, 430030, China.

^*^Corresponding authors

E-mail addresses: wangyanchao@scu.edu.cn (Y. Wang), hongtan@scu.edu.cn (H. Tan), [tinglanpathology@gmail.com](mailto:tinglanpathology@gmail.com) (T. Lan)

^1^These authors contributed equally to this work.

**Characterization of adhesive patches**

The attenuated total reflection Fourier transform infrared spectroscopy (ATR-FTIR) scans of the top and bottom sides of the patch were conducted using an infrared spectrometer, with a scanning frequency of 16 times and a resolution of 4 cm⁻¹. The mechanical properties of the adhesive patch were tested using a universal testing machine. The adhesive patch was cut into dumbbell-shaped strips with a standard width of 2 mm using a dumbbell-shaped cutter. The strips were fixed with fixtures and the stretching speed was 150 mm/min. The breaking strength and elongation at break of the strips were recorded. The bonding strength of the adhesive patch was tested using the shear bonding method on the universal testing machine. The adhesive patch was adhered to a base material such as pig skin, with an overlapping area of 15 × 25 mm, and the stretching speed was 50 mm/min.

**Coarse-Grained Molecular Dynamics Simulation**

Due to the large scale involved in film curling, coarse-grained molecular dynamics (CGMD) simulations were employed to study the behavioral changes of films upon exposure to water[1]. Using the GROMACS software package and visualizing with visualized molecular dynamics (VMD), CGMD simulations were performed to investigate the behavior of film in aqueous environments under the Martini 3.0 force field. After mapping the PU molecule according to bead types (Fig. S1), the bond length and angle parameters for PU were fitted using these bead types[2, 3].

**Fig. S1.** Chemical structure of PU and corresponding mapping scheme.

To obtain the topological file of the coarse-grained PU molecule, it is necessary to fit the bond length and bond angle parameters from the all-atom trajectory. The specific simulation process is as follows: The GAFF force field is adopted, and 20 identical monomers are placed in a box with an initial volume of 8 nm³. Water molecules are modeled using the TIP3P model. The Berendsen method is used to maintain pressure, with a compressibility of 4.5×10⁻⁵ bar⁻¹, and the pressure coupling is set to isotropic. The temperature is maintained at 300 K using the v-rescale algorithm with a time constant of 0.1 ps. The cutoff values for electrostatic and van der Waals interactions are set to 1.0 nm. Verlet is used to handle short-range non-bonded interactions, and the LINCS algorithm is employed to constrain bond lengths.

The non-scientific molecular conformations were removed using the steepest descent algorithm, and the system energy was minimized for 5000 steps. Then, a rapid compression of 200 ps at 100 bar in the NPT ensemble was performed. A 10 ns annealing process was carried out in the NVT ensemble. During the annealing process, the temperature changed periodically within the range of 300 - 500 K, with a balance cycle of 1 ns. Subsequently, a 100 ns equilibrium was conducted in the NPT ensemble at 1 bar and 300 K to achieve a volume density close to the experimental condition. Finally, from the sampling trajectories generated in the 100 ns NPT ensemble, the bond lengths and angles were fitted, with a step size of 2 fs. The coarse-grained bond length and bond angle fitting distributions were found to be in good agreement with the full-atom results, and the specific bond length and angle information can be found in Tables S1 and S2.

**Table S1.** Bond length parameter.

|  | Bond length (nm) | k (kJ/mol/nm) |
| --- | --- | --- |
| P2-C2 | 0.22 | 15160 |
| P2-C1 | 0.44 | 10000 |
| C2-C3 | 0.48 | 1700 |
| C2-N4 | 0.38 | 45000 |
| C3-N4 | 0.32 | 90000 |
| C3-P2 | 0.18 | 90000 |
| N4a-C1 | 0.412 | 5000 |
| P2-N4 | 0.39 | 3582 |
| P2-TN2a | 0.32 | 17640 |
| TN2a-N2q | 0.30 | 8137 |
| N2q-P2 | 0.41 | 7718 |
| SN3r-SN3r | 0.36 | 7000 |
| N4-N4 | 0.30 | 4823 |
| N4-SP2 | 0.30 | 5029 |
| C1-C1 | 0.49 | 4202 |

**Table S2.** Bond Angle parameter.

|  | Bond angle (°) | k (kJ/mol/rad^2^) |
| --- | --- | --- |
| C2-P2-C1 | 155 | 150 |
| P2-C2-C3 | 115 | 175 |
| P2-C2-N4 | 154 | 200 |
| C3-C2-N4 | 55 | 4500 |
| C2-C3-N4 | 75 | 1300 |
| N4-C3-P2 | 144 | 300 |
| C2-N4-C3 | 48 | 5000 |
| C3-P2-C1 | 155 | 150 |
| P2-C1-N4a | 128 | 24 |
| C1-N4a-C1 | 129 | 50 |
| N4a-C1-N4a | 170 | 50 |
| SC3-P2-N4 | 174 | 120 |
| SC3-SC3-P2 | 125 | 387 |
| SC3-P2-TN2a | 140 | 72 |
| P2-N4-N4 | 115 | 40 |
| P2-N4-SP2 | 102 | 149 |
| N4-N4-SP2 | 134 | 32 |
| P2-TN2a-N2q | 108 | 71 |
| TN2a-N2q-TN2a | 103 | 37 |
| TN2a-N2q-P2 | 89 | 23 |
| N2q-P2-C1 | 136 | 35 |
| TN2a-P2-SC3 | 140 | 72 |
| P2-SN3r-SN3r | 109 | 50 |
| SN3r-SN3r-SN3r | 123 | 80 |
| SC3-P2-SN3r | 162 | 180 |

In order to further verify the scientificity of the fitted bond length and bond angle parameters, the solubility accessible surface area (SASA), mean square rotational radius (Rg), and end-to-end distance of the molecules of PU in solution and in the dry state were compared. The final results indicated that the coarse-grained molecular conformation could maintain a good consistency with the full-atom model under various conditions, and could be used for further analysis. After obtaining the scientific coarse-grained structure and topology file of PU, the simulation of the bending process of the PU/PEG mixed membrane when placed in water began. First, 250 PU molecules (red) and 1547 PEG molecules (blue) were compressed to obtain the PU/PEG mixed membrane. By observing the component density distribution in the Z direction (Fig. S2), it was found that the hydrophilic PEG was mainly distributed at the bottom of the membrane. Then, this membrane was placed in an environment filled with water droplets to simulate the non-equilibrium experimental phenomenon of the membrane being placed in water.


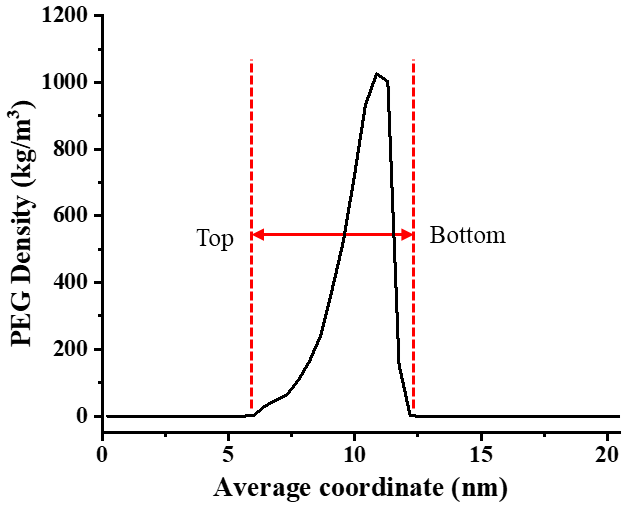


**Fig.S2.** Component density distribution of PEG in the Z direction.

**Macrophage polarization *in vitro***

Rat Schwann cells (RSC96) and murine macrophage-like RAW264.7 cells were purchased from the Cell Bank of the Chinese Academy of Sciences (Shanghai, China). Cells were cultured in high-glucose Dulbecco’s modified Eagle’s medium (DMEM) supplemented with 10% fetal bovine serum (FBS; Gibco, USA), 100 U/mL penicillin, and 100 μg/mL streptomycin (Gibco, USA). Cultures were maintained at 37 °C in a humidified incubator with 5% CO₂ (Thermo Fisher Scientific, USA) and passaged when reaching 80–90% confluence. Cells at passage 3 were used for all subsequent experiments.

RAW264.7 macrophage-like cells were used to evaluate the anti-inflammatory performance of PU-based patches. The experiment included three groups: control (no patch), PU patches, and PU/PGA_10_ patches. Patches were pre-placed on the bottom of 24-well plates prior to cell seeding. Cells were seeded at 1×10^5 cells per well and allowed to attach for 12 h. Subsequently, the cultures were stimulated with lipopolysaccharide (LPS; Sigma, USA) at a final concentration of 100 ng/mL for either 8 h or 24 h. The 8-h time point was used for assessing inflammatory gene expression at the mRNA level, whereas the 24-h time point was used to quantify cytokine release in the culture supernatants. Macrophage responses were evaluated by assessing inflammation-related transcripts using reverse transcription quantitative polymerase chain reaction (RT-qPCR) and by determining cytokine release in the conditioned medium via enzyme-linked immunosorbent assay (ELISA). Briefly, total RNA was isolated from the treated cells using a Cell Direct RT-qPCR Kit (Foregene, Chengdu, China). Approximately 1 μg of RNA was reverse-transcribed with the PrimeScript RT Reagent Kit (Takara, Japan). Real-time PCR was carried out using TB Green Premix (Takara, Japan), and gene expression was normalized to GAPDH and calculated with the 2^−ΔΔCt^ approach. Transcripts associated with M1 polarization (TNF-α) and M2 polarization (IL-10) were quantified; primer information is provided in Table S4. Levels of TNF-α and IL-10 in the supernatants were measured using commercial ELISA kits (Dakewe Bio-engineering Co., Ltd., Shenzhen, China) following the manufacturer’s protocols.
**The degradation performance of the adhesive patch**

Cut the PU/PGA_10_ patches into 1 cm × 1 cm square pieces and place them separately in 15 mL centrifuge tubes. Add 10 mL of PBS solution containing lipase. Place the centrifuge tubes in a 37℃ shaking incubator and shake them. Replace the degradation solution once a week. Take the patches at different times and wash the surface with PBS to remove residual enzymes and impurities. Dry the patches in an oven and weigh them to calculate the degradation rate. The initial mass of the adhesive is m_0_, and the mass after degradation at different times is m_t_. The degradation rate calculation formula is:

$$\text{Degradation rate(\%)=}\frac{\text{m}_{\text{0}}\text{-}\text{m}_{\text{t}}}{\text{m}_{\text{0}}}\text{×100\%}$$

Healthy SD rats (female, weighing 180 - 220 g) were anesthetized, and the surface hair on their backs was removed using a hair removal tool. The backs were disinfected with iodophor and the skin was opened. The irradiation-sterilized PU/PGA_10_ patches (5 mm × 5 mm) were implanted subcutaneously. The wounds were then sutured, and the rats were disinfected with iodophor. The rats were sacrificed at different times, and the back skin was peeled off to observe the degradation of the patches in the body.

**Fig. S3.** Synthetic route of WPU emulsion.


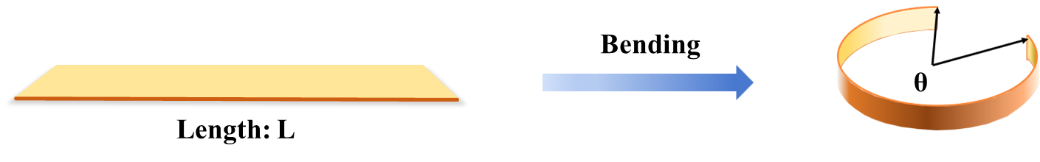


**Fig. S4**. Schematic diagram of the calculation of self-curling curvature.


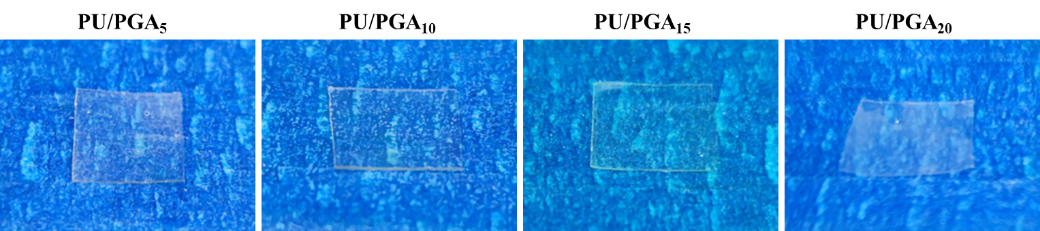


**Fig. S5.** Shape of PU/PGA_X_ film before water soaking.


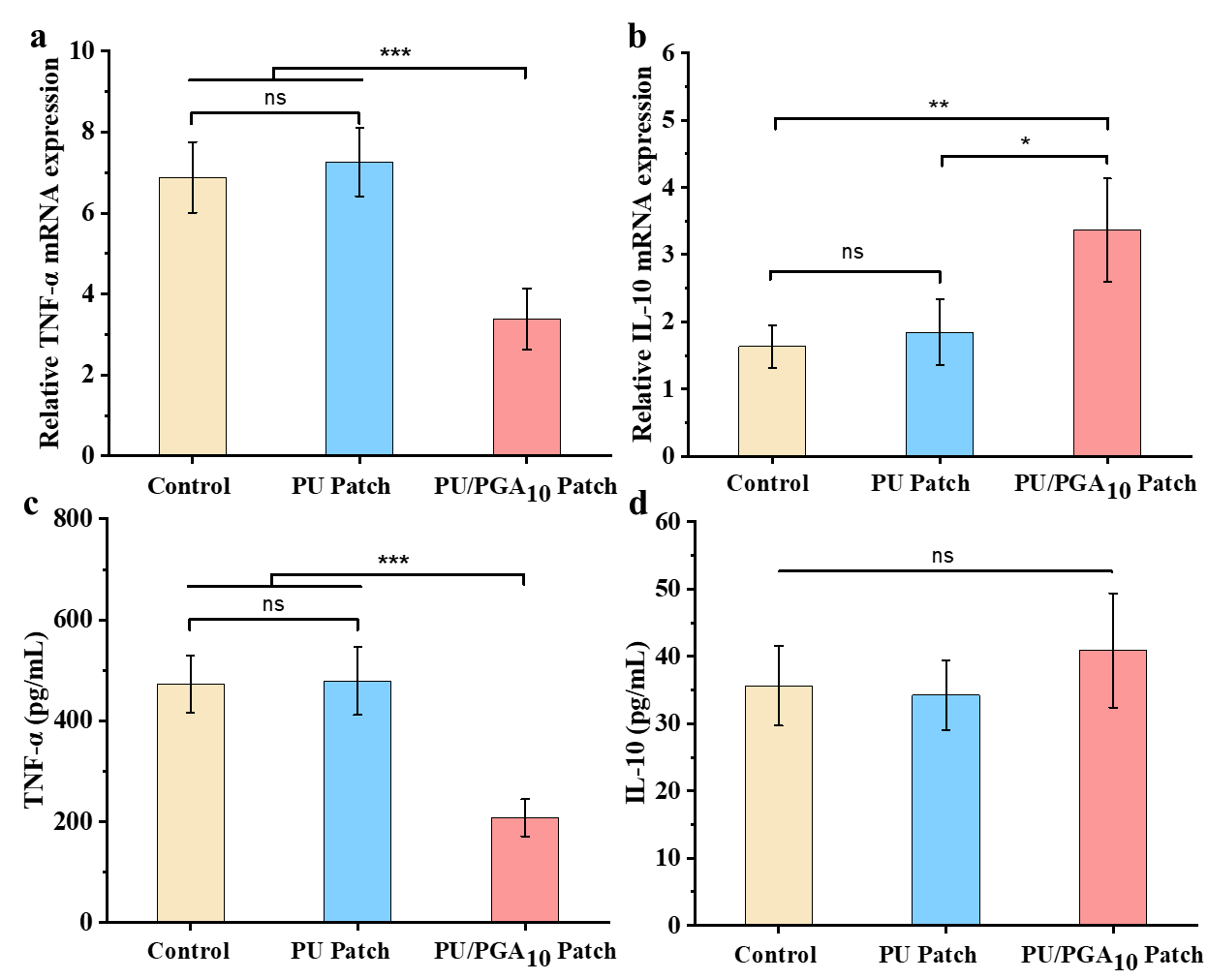


**Fig. S6.** RT-qPCR measurement of (a) TNF-α and (b) IL-10 expression in macrophages cultured with the patch, GAPDH as an internal control; ELISA assay of (c) TNF-α and (d) IL-10 for macrophages cultured with the patch.


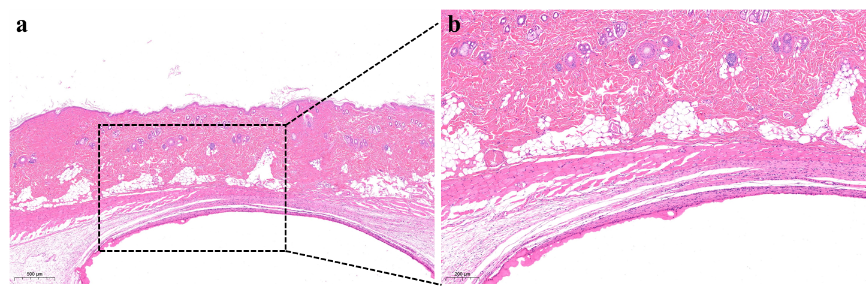


**Fig. S7.** (a) H&E-stained sections of the implanted PU/PGA_10_ patch together with surrounding skin tissue; (b) partial enlarged detail.


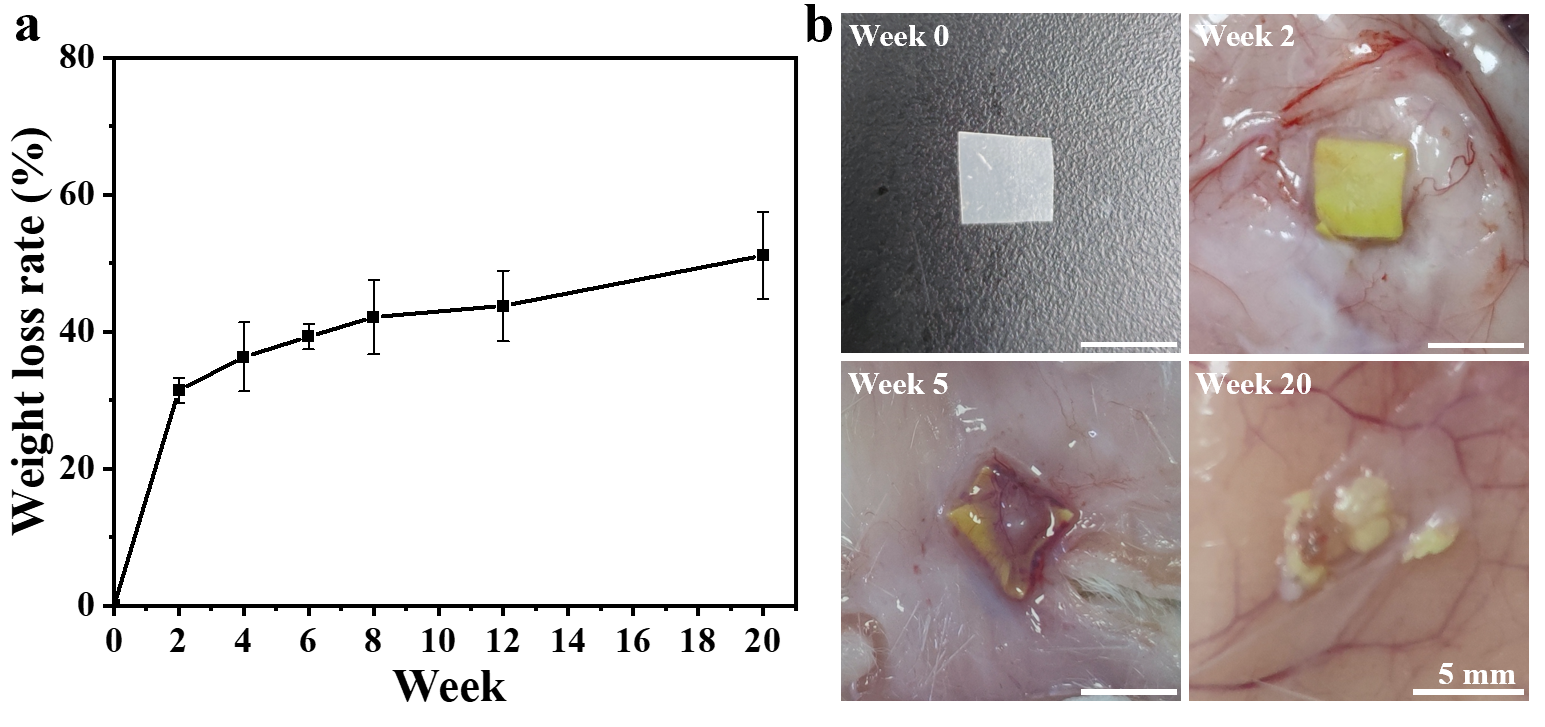


**Fig. S8.** (a) Enzymatic degradation curve of PU/PGA_10_ patch in vitro; (b) Subcutaneous degradation diagram of PU/PGA_10_ patch in rats *in vivo*.


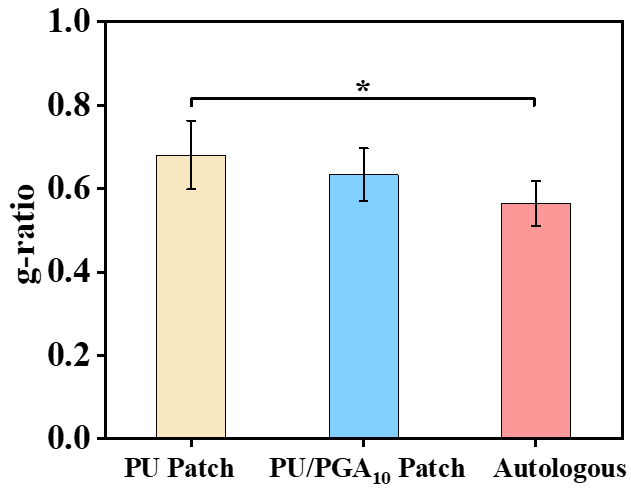


**Fig. S9.** G-ratio of the optimal myelination index of the regenerated nerve at 10 weeks after surgery.


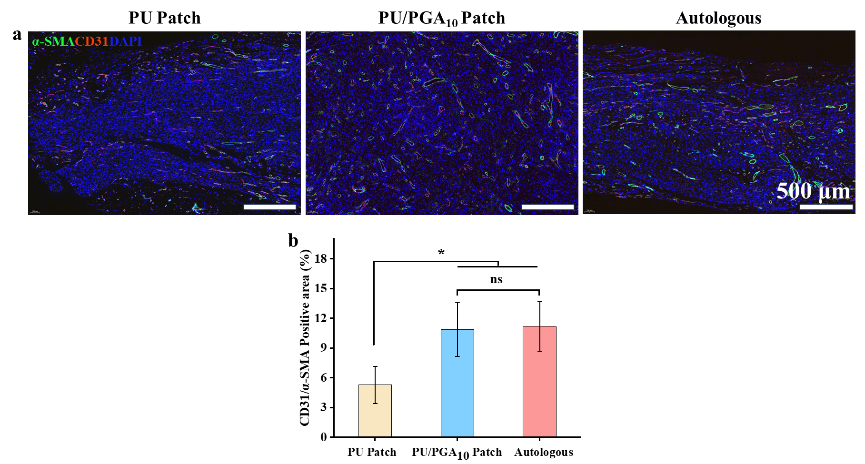


**Fig. S10.** (a) Immunofluorescence staining of CD31 and α-SMA and (b) percentage of co-localized positive at 10 weeks after surgery.


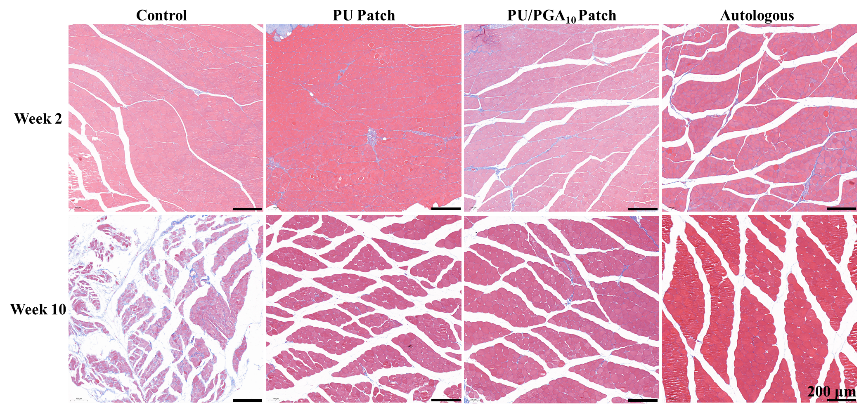


**Fig. S11.** Masson staining of gastrocnemius muscle in each group at 2 and 10 weeks after operation.

**Table S3**. Atomic percentages in the XPS spectra at the top and bottom of PU film and PU/PGA_10_ film.

| Samples | Surface atomic percentages | | | | | Oxygen components | |
| --- | --- | --- | --- | --- | --- | --- | --- |
|  | C | N | O | N^+^ | O=C | O-C | O=C |
| PU+ | 72.89 | 5.44 | 21.67 | 1.08 | 6.14 | 71.66 | 28.34 |
| PU- | 69.73 | 2.62 | 27.66 | 0.37 | 2.21 | 92.01 | 7.99 |
| PU/PGA_10_+ | 69.86 | 4.97 | 25.17 | 1.07 | 4.46 | 82.27 | 17.73 |
| PU/PGA_10_- | 72.83 | 4.45 | 22.72 | 0.85 | 5.46 | 75.97 | 24.03 |

**Table S4**. Primer sequences of RT-qPCR for *in vitro* cell study.

| 8Primer | Sequence (5' to 3') |
| --- | --- |
|  |  |
| GAPDH-f | CATGGCCTTCCGTGTTCCTA |
| GAPDH-r | GCCTGCTTCACCACCTTCTT |
| TNFα-f | CAGGCGGTGCCTATGTCTC |
| TNFα-r | CGATCACCCCGAAGTTCAGTAG |
| IL10-f | TCCCGAACATCGACAGCCCCA |
| IL10-r | AGGGGCAGTATCTTGCACCAGG |

**References**

[1] A. Hochwallner, J. Stampfl, A Martini 3 coarse-grain model for the simulation of the photopolymerizable organic phase in dental composites, RSC Advances 12(19) (2022) 12053-12059. <https://doi.org/10.1039/d2ra00732k>.

[2] P. Vainikka, S. Thallmair, P.C.T. Souza, S.J. Marrink, Martini 3 Coarse-Grained Model for Type III Deep Eutectic Solvents: Thermodynamic, Structural, and Extraction Properties, ACS Sustainable Chemistry & Engineering 9(51) (2021) 17338-17350. <https://doi.org/10.1021/acssuschemeng.1c06521>.

[3] R. Alessandri, J. Barnoud, A.S. Gertsen, I. Patmanidis, A.H. de Vries, P.C.T. Souza, S.J. Marrink, Martini 3 Coarse‐Grained Force Field: Small Molecules, Advanced Theory and Simulations 5(1) (2021). <https://doi.org/10.1002/adts.202100391>.
